# The spliceosome component SNRPC promotes glioma progression by sustaining mitochondrial function and TNFAIP2 signaling

**DOI:** 10.1038/s41419-026-09065-6

**Published:** 2026-07-17

**Authors:** Peng Ma, Jiang Wu, Zhen Ma, Kai Xu, Xingqi Wang, Kun Yuan, Ya Peng

**Affiliations:** 1 Department of Neurosurgery, The Third Affiliated Hospital of Soochow University, Changzhou, China; 2 Department of Neurosurgery, Taicang Loujiang New City Hospital, Suzhou, China; 3 Department of Neurosurgery, The First Affiliated Hospital of Soochow University, Suzhou, China; 4 Department of Neurosurgery, The First People’s Hospital of Changzhou, Changzhou, China

**Keywords:** CNS cancer, Oncogenes

## Abstract

Human glioma is a devastating primary brain tumor with a dismal prognosis, necessitating the identification of novel therapeutic targets. The small nuclear ribonucleoprotein C (SNRPC), a core spliceosome component, is implicated in cancer, but its role in glioma remains unexplored. This study aimed to delineate the expression, function, and underlying mechanisms of SNRPC in human glioma. The bioinformatic analysis revealed that *SNRPC* is significantly upregulated in glioma tissues, with expression levels correlating strongly with higher tumor grade, aggressive molecular subtypes (IDH wild-type, 1p/19q non-codeleted), and poor patient prognosis, establishing it as a robust independent biomarker. Single-cell analysis pinpointed *SNRPC* enrichment in malignant glioma cells, and co-expression studies linked it to mitochondrial metabolism and oxidative phosphorylation. SNRPC expression is also upregulated in locally-resected glioma tissues and various glioma cell types. Functionally, silencing (by targeted shRNA) or knocking out (via CRISPR/Cas9 method) of SNRPC profoundly attenuated glioma cell proliferation, migration, and invasion, while inducing G1-S arrest and apoptosis. These effects were specifically observed in malignant glioma cells, sparing normal astrocytes. Mechanistically, loss of SNRPC led to severe mitochondrial dysfunction, characterized by impaired mitochondrial respiration, ATP depletion, membrane depolarization, and excessive ROS (reactive oxygen species) production. Conversely, SNRPC overexpression enhanced mitochondrial bioenergetics and promoted malignant phenotypes. SNRPC promoted glioma malignancy by regulating the expression of a key oncogene tumor necrosis factor alpha-induced protein 2 (TNFAIP2), which acts as a key downstream effector to drive increased cell proliferation and migration. Critically, SNRPC knockdown suppressed subcutaneous glioma xenograft growth and disrupted mitochondrial bioenergetics. Its knockout also impeded the intracranial glioma growth in an orthotopic mouse model. Our findings establish SNRPC as a pivotal driver of glioma malignancy by sustaining mitochondrial hyperfunction and TNFAIP2 expression essential for tumor cell proliferation.

## Introduction

Human glioma, the most prevalent and aggressive primary malignant brain tumor in adults [1, 2], presents a formidable clinical challenge [3, 4]. Despite a multimodal treatment paradigm of surgical resection, radiotherapy, and chemotherapy, the prognosis for patients with glioma, particularly glioblastoma (GBM), remains dismal, with a median survival of less than two years [5, 6]. The therapeutic efficacy of the current standard of care is severely constrained by the tumor’s diffuse infiltrative growth, inherent chemo- and radioresistance, and near-universal recurrence [1, 7]. These obstacles highlight an urgent need to elucidate the fundamental molecular mechanisms driving glioma pathogenesis to develop more effective therapeutic interventions [8–10].

The advent of targeted therapies, which aim to selectively inhibit dysregulated molecular pathways in tumor cells, has introduced a promising new approach to glioma treatment [11–14]. Although these agents offer the potential for greater precision and reduced toxicity compared to conventional chemotherapy, their clinical utility has been modest [11–14]. The profound intra- and inter-tumoral heterogeneity of glioma, along with the rapid emergence of acquired resistance, frequently undermines therapeutic efficacy [11–14]. Consequently, the identification and validation of novel molecular targets that are indispensable for glioma sustenance and progression are critical for overcoming the present therapeutic stalemate and improving patient outcomes [11–14].

The small nuclear ribonucleoprotein C (SNRPC) is an integral component of the U1 small nuclear ribonucleoprotein (snRNP) [15–17], a cornerstone of the spliceosome machinery [15, 16]. Functionally, SNRPC assumes a pivotal role in pre-mRNA splicing through its recognition of the 5′ splice site, which is a determinative step in the precise excision of introns [15, 16]. By modulating alternative splicing, SNRPC influences the expression of a vast repertoire of gene isoforms, thereby regulating fundamental cellular processes. Indeed, dysregulation of the splicing machinery is increasingly implicated in human diseases, particularly cancer [18–21], where aberrant splicing events can generate oncogenic protein variants that drive tumorigenesis [18–21].

Although the broader role of aberrant splicing in cancer is increasingly appreciated, the specific contribution of SNRPC to tumorigenesis remains largely unexplored. In this study, we tested the expression, biological function, and underlying molecular mechanisms of SNRPC in human glioma. Our results demonstrate that SNRPC is overexpressed in glioma and that this upregulation promotes malignant phenotypes. These findings establish SNRPC as a putative driver of glioma progression and identify it as a promising new candidate for therapeutic targeting.

## Materials and methods

### Reagents and antibodies

A comprehensive panel of fluorescent probes was procured from Thermo-Fisher Invitrogen (Suzhou, China), including reagents for assessing apoptosis (TUNEL), cell proliferation (EdU), cellular and mitochondrial reactive oxygen species (MitoSOX, DCF-DA), and mitochondrial membrane potential (JC-1). Primary and secondary antibodies for immunoblotting and immunohistochemistry were sourced from Cell Signaling Technology (Danvers, MA) and Abcam (Cambridge, United Kingdom). All small hairpin RNA (shRNA), CRISPR/Cas9 knockout, and overexpression viral constructs, were synthesized and provided by Genechem (Shanghai, China). General cell culture reagents, including fetal bovine serum (FBS), Dulbecco’s Modified Eagle Medium (DMEM), and antibiotic solutions, were supplied by Hyclone (Logan, UT). All other chemical reagents, such as N-acetyl cysteine (NAC) and D-glucose, were of analytical grade and procured from Sigma-Aldrich (St. Louis, MO).

### Human tissue specimens and cells

This study utilized human tissues obtained with prior informed consent from all participants. A cohort of sixteen (*n* = 16) patients undergoing surgical resection for primary high-grade glioma (HGG) contributed both tumor tissue and matched, adjacent non-neoplastic brain tissues, as previously described [22–24]. These normal tissues consist of very small amounts of adjacent non-neoplastic brain tissue that were inevitably attached to the periphery of the surgically resected tumor mass during surgical resection. The primary patient-derived glioma cell cultures (designated P1, P2, and P3) and primary human astrocyte cultures (Astrocytes1 and Astrocytes2, derived from patients P1 and P2, respectively) were generously provided by Dr. Cao [22–24]. The immortalized human glioma cell lines A172 was also obtained from this source. The primary glioma cells (P1-P3) used throughout this study were molecularly characterized as isocitrate dehydrogenase (*IDH*)-wildtype GBM cells exhibiting *PTEN* deletion, *EGFR* amplification, and *p53*-wildtype status, and were maintained in DMEM supplemented with 10% FBS. This growth medium also contained epidermal growth factor (EGF, 25 ng/mL), basic fibroblast growth factor (bFGF, 25 ng/mL), and heparin (2 μg/mL). The primary human astrocytes and A172 cells were cultured in standard DMEM supplemented with 10% FBS. All cells were maintained in a humidified incubator at 37 °C with a 5% CO₂ atmosphere. All experimental procedures involving human-derived materials were conducted in strict adherence to the principles outlined in the Declaration of Helsinki and received formal approval from the Ethics Board of Soochow University (SUDA20230203A15).

### Primary cortical pyramidal neurons and immunofluorescence staining

Primary cortical pyramidal neurons were established from layer V of the sensorimotor cortex dissected from E18.5 mouse embryos. Dissected tissues were minced and subsequently dissociated enzymatically using trypsin-EDTA. Following trypsin inactivation, the cellular suspension was gently triturated to obtain a single-cell preparation. Cells were then seeded onto poly-D-lysine-coated culture dishes at a density of 10,000 cells/cm². Cultures were maintained at 37 °C in a humidified incubator in Neurobasal medium supplemented with B-27, GlutaMAX, and penicillin-streptomycin. Fifty percent of the culture medium was exchanged every 2 days. For immunofluorescence analysis, cultured neurons were fixed with 4% paraformaldehyde for 10 min, followed by three washes with phosphate-buffered saline (PBS). Cells were then permeabilized and blocked for 1 h in a solution containing 0.3% Triton X-100 and 5% goat serum. Primary anti-MAP2 antibody incubation was performed overnight at 4 °C. After three subsequent PBS washes to remove unbound primary antibody, appropriate secondary antibodies were applied for 2–3 h at room temperature.

### Western blots

Total cellular or tissue proteins were first extracted in a suitable lysis buffer (Biyuntian, Wuxi, China), and protein concentrations precisely determined using a BCA protein assay kit (Thermo-Fisher Scientific). For each sample, an equivalent mass of protein (typically 20–30 µg) was denatured and fractionated by size via sodium dodecyl sulfate-polyacrylamide gel electrophoresis (SDS-PAGE) on 8–12% gels. The resolved proteins were subsequently transferred onto a polyvinylidene difluoride (PVDF) membrane by overnight electroblotting at 4 °C. Following transfer, membranes were blocked to prevent non-specific antibody binding, followed by sequential incubation with target-specific primary antibodies, and incubation with the appropriate horseradish peroxidase (HRP)-conjugated secondary antibodies, both performed at 4 °C. The resulting immunoreactive bands were visualized using an enhanced chemiluminescence (ECL) substrate (Amersham ECL Plus). Full, uncropped images of all blots are provided in the Supplementary Information (Fig. S1).

### Quantitative real-time PCR (qRT-PCR)

Total RNA was isolated from cell or tissue samples using the TRIzol reagent, and its purity and concentration were verified spectrophotometrically with a Nanodrop 2000 instrument. A standardized amount of total RNA was then reverse-transcribed into complementary DNA (cDNA) using the Reverse Transcriptase system (Promega). The resulting cDNA served as the template for qPCR, which was performed using the SYBR Green PCR Master Mix (Thermo Fisher Scientific) on a compatible real-time thermal cycler. The relative quantification of gene expression was calculated using the 2^−ΔΔ^Cq method, where all target gene expression levels were normalized to the expression of the stably expressed housekeeping gene, *GAPDH*, which served as the internal reference control. The primers were provided by Biyuntian (Wuxi, China).

### Stable SNRPC silencing via lentiviral shRNA

To achieve stable knockdown of SNRPC, lentiviral vectors from the GV369 construct series (Genechem, Shanghai, China) were employed to deliver short hairpin RNAs (shRNAs). For the silencing of *SNRPC*, glioma cells or astrocytes were transduced with lentiviral particles encoding one of two distinct shRNA sequences: shSNRPC-Sq1 (targeting: 5′-*AAGAGAATGTGAAAGACTATTATCAGA*-3′) and shSNRPC-Sq2 (targeting: 5′-*GCTGCATTTCAACAAGGAAAGATACCT*-3′). Transductions were performed at a multiplicity of infection (MOI) of 10 for 48 h. Subsequently, stably transduced cells were selected and maintained by continuous culture in puromycin (1.5 µg/mL)-containing medium for a minimum of five passages. A parallel control cells was generated by transduction with a lentivirus encoding a non-targeting scramble shRNA (shC). The efficacy of gene silencing in all resulting stable cell populations was rigorously validated at both the mRNA and protein levels.

### CRISPR/Cas9-mediated SNRPC knockout

The genetic knockout of *SNRPC* was accomplished using a CRISPR/Cas9-based strategy. Glioma cells were infected with lentiviral constructs co-expressing *Streptococcus pyogenes* Cas9 (from a vector provided by Dr. Cao [22–24]) and one of one single-guide RNA (sgRNA) designed to target the human *SNRPC* gene (targeting: *GGTGGTATCATCGCCCCTGC*, PAM: *AGG*). Following infection, the mixed cell population underwent five passages of selection with puromycin (1.5 µg/mL). To isolate knockout cell colonies, cells were then seeded into 96-well plates at limiting dilution. The successful knockout of SNRPC protein in the resulting clones was confirmed by immunoblotting, leading to the establishment of two stable knockout cell colonies (designated ‘koSNRPC-C1’ and ‘koSNRPC-C2’). The knockout control cells (‘koC’) were concurrently generated by transducing cells with a lentiviral construct expressing Cas9 and a non-targeting scramble sgRNA.

### Stable overexpression of SNRPC

For experiments requiring ectopic gene expression, glioma cells were transduced with a lentiviral vector from the tag-free GV248 (Genechem, Shanghai, China). This vector is engineered to express the full-length cDNA sequence ofr human *SNRPC*. After transduction, stable cell populations were established through selection with puromycin (1.5 µg/mL) over five passages. The resulting two stable cell selections, designated as ‘oeSNRPC-Sl1’ and ‘oeSNRPC-Sl2’, were validated for robust overexpression at both the transcript and protein levels. A corresponding control cell selection (termed ‘Vec’) was generated by transduction with the empty GV248 vector.

### Quantification of mitochondrial complex I activity and cellular ATP

The enzymatic activity of mitochondrial ETC (electron transport chain) Complex I was determined using a commercially available kit (Sigma-Aldrich), following the manufacturer’s specifications [25–27]. This assay spectrophotometrically measures the rate of NADH oxidation to NAD+, a direct readout of ETC Complex I activity and function, by monitoring the decrease in absorbance at 435 nm. Cellular ATP levels were quantified using a colorimetric ATP assay kit (Sigma-Aldrich) according to the provided protocols. For both assays, analyses were performed on 20 µL aliquots of cellular or tissue homogenate, standardized to a total protein content of 20 µg.

### Determination of glutathione state

The cellular glutathione redox status was evaluated by quantifying the ratio of reduced glutathione (GSH) to oxidized glutathione (GSSG) with a dedicated assay kit (Thermo-Fisher Scientific, Suzhou, China). In this procedure, lysates were incubated in a reaction mixture containing 5,5′-dithio-bis (2-nitrobenzoic acid) (DTNB), glutathione reductase, and NADPH. The kinetic formation of the chromophore was monitored spectrophotometrically at 425 nm over a 5-min period. Absolute concentrations of GSH and GSSG were calculated by referencing a standard curve generated from known concentrations of each species. Each measurement included 20 µL aliquots of cellular or tissue homogenate, standardized to a total protein content of 20 µg.

### Assessment of lipid peroxidation

Lipid peroxidation was quantified via a thiobarbituric acid reactive substances (TBAR) assay (Thermo-Fisher Scientific, Suzhou, China). Protein lysates from cells or tissues were reacted with thiobarbituric acid (TBA) to form the TBAR adduct. Following a cold incubation and centrifugation step to remove precipitates, the optical density of the resulting supernatant was measured at 525 nm. Each measurement included 20 µL aliquots of cellular or tissue homogenate, standardized to a total protein content of 20 µg.

### Measurement of oxygen consumption rate (OCR)

Cellular respiratory function was assessed by measuring the oxygen consumption rate (OCR) with an Agilent Seahorse XF24 Extracellular Flux Analyzer, adhering to established methodologies [28]. A standard mitochondrial stress test was performed by the sequential injection of metabolic inhibitors: 1 µM oligomycin (to inhibit ATP synthase), 0.5 µM FCCP (to uncouple the proton gradient and induce maximal respiration), and a final mixture of 0.5 µM antimycin A and 0.5 µM rotenone (to inhibit Complexes III and I, respectively). This protocol allowed for the calculation of basal respiration, ATP-linked respiration, maximal respiratory capacity, and non-mitochondrial oxygen consumption. All OCR values were normalized to the total protein content of the corresponding well.

### Cell viability, clonogenic and death assays

Cell viability was determined using the Cell Counting Kit-8 (CCK-8) assay. Genetically engineered glioma cells or astrocytes were seeded into 96-well plates at a density of 3000 cells per well. After a 96-h incubation period, the CCK-8 reagent was added, and the plates were incubated for an additional 100 min before measuring the absorbance at 450 nm with a microplate reader. For the clonogenic (colony formation) assay, cells were seeded at a low density from a single-cell suspension into 6-well plates containing complete growth medium. Following a 12-day incubation at 37 °C and 5% CO₂, the resulting colonies were fixed, stained, and enumerated. Cell death was measured via Trypan blue staining assays. Both viable (clear, unstained) and non-viable (blue-stained) cells were counted in the designated counting grids. The percentage of cell death was subsequently calculated by dividing the number of blue-stained cells by the total number of cells counted.

### Fluorometric quantification of Caspase-3 activity

The caspase-3 activity was determined via a fluorometric kinetic assay. Brief, an aliquot of each lysate, containing precisely 20 µg of total proteins, was introduced into a reaction buffer containing the fluorogenic substrate Ac-DEVD-AMC (Sigma-Aldrich). The proteolytic liberation of the fluorescent aminomethylcoumarin (AMC) moiety by active caspase-3 was continuously monitored using a microplate reader, with fluorescence measured at an excitation/emission wavelength pair of 375/450 nm.

### Fluorescence staining

For cytological analyses using fluorescent probes, cells were cultured on glass coverslips within 24-well plates at an initial density of 3 × 10⁴ cells per well for the duration specified by each experiment. Cells were then washed with PBS before being incubated with the designated fluorescent probe according to its optimized protocols. Qualitative imaging and morphological assessment were performed on a Leica fluorescence microscope. For quantitative analysis of signal intensity, parallel samples were analyzed on a Hitachi F-7000 spectrophotometer.

### Transwell assays

The in vitro migratory and invasive capacities of the glioma cells were rigorously assessed using a Boyden chamber (Transwell) assay (Corning, Suzhou, China). To evaluate migration, a cell suspension in serum-free medium was placed in the upper compartment of the Transwell insert. The lower chamber was filled with medium supplemented with 10% FBS, establishing a chemoattractant gradient. After 24 h, cells that had not traversed the porous membrane were mechanically removed from the upper surface. Cells that had successfully migrated to the lower surface were then fixed, stained with crystal violet, and quantified under microscopy. To assess in vitro invasion, the identical protocol was employed, with the critical modification of pre-coating the Transwell insert membrane with a basement membrane matrix (Matrigel), thereby requiring cells to actively degrade this barrier to traverse the pores.

### Chromatin Immunoprecipitation (ChIP) assay

The physical recruitment of RNA Polymerase II (Pol II) to the *TNFAIP2* promoter was evaluated via ChIP assay. Specific protein-DNA complexes were isolated by executing immunoprecipitation with a high-affinity anti-Pol II antibody (Abcam, Shanghai, China). DNA was cross-linked using 1% formaldehyde for 10 min at room temperature to stabilize protein-DNA interactions, followed by quenching with 0.125 M Glycine. To obtain suitable chromatin fragments, sonication was carried out, yielding genomic DNA fragments predominantly between 200 and 500 bp in length. The fraction of the *TNFAIP2* promoter physically associated with Pol II was quantified using real-time PCR with verified primers (Forward primer: TGACTCTGCGACTATTTCAGGC, Reverse primer TGGAAGCGGAGGTCACTTAC). These primers were specifically designed to target the binding motifs situated within the region to the Transcription Start Site (TSS) of *TNFAIP2*.

### siRNA

For the siRNA-mediated silencing of SNRP components, P1 primary glioma cells were seeded in 6-well plates and cultured to reach 50% confluence before undergoing a dual-transfection protocol to ensure robust knockdown. Specifically, cells were transfected with 200 nM of siRNAs targeting SNRPA (targeting CCAAGACCGACUCAGAUAU), SNRPB (targeting CAAGCCAAAGAACUCCAAA), SNRPC (targeting GGACCAUAUUGCCAAGUUA), SNRPD1 (targeting GCAUCAGUUGCUACCUAAA), or SNRPE (targeting GCUCUAUGAGCAAGUGAAU), or a non-targeting control (siC, UAAGGCUAUGAAGAGAUAC), using Lipofectamine 3000 (Invitrogen). To maintain sustained gene silencing, a second round of transfection was performed at the 24-h mark using the same siRNA concentration and reagents. The cells were then incubated for an additional 24 h, totaling a 48-h treatment period, before being harvested for quantitative RT-PCR (qRT-PCR) to verify knockdown efficiency, or further maintained for functional assessments including CCK-8 viability assays at 96 h and EdU proliferation assays at 60 h post-second transfection.

### The subcutaneous and orthotopic glioma xenograft establishment

All in vivo experiments were conducted using a cohort of 5- to 6-week-old e athymic nude mice (half male and half female, body weight 18.0–18.4 g). Animals were housed in specific-pathogen-free (SPF) conditions within individually ventilated cages, with unrestricted access to sterile food and water. For subcutaneous xenograft model, each mouse was injected subcutaneously with five million P1 glioma cells in the right flank. To establish intracranial tumors, surgical procedures were performed under general anesthesia induced by an intraperitoneal (IP) administration of ketamine (100 mg/kg) and xylazine (10 mg/kg). Following previously validated stereotactic protocols [23, 29, 30], P1 patient-derived glioma cells were precisely injected into the brain. Post-operatively, mice were carefully monitored until they regained full consciousness. Tumor engraftment and subsequent growth were monitored non-invasively using Magnetic Resonance Imaging (MRI). To mitigate experimental bias, animals were randomly assigned to treatment groups, and all subsequent data collection and analyses were performed in a blinded fashion. At the designated experimental endpoint, mice were humanely euthanized by CO₂ asphyxiation followed by cervical dislocation for tissue harvesting. All animal procedures were performed in strict accordance with protocols approved by the Institutional Animal Care and Use Committee (IACUC) and the Animal Ethics Review Board of Soochow University (SUDA20230203A15).

### Immunohistochemical (IHC) staining

IHC analysis was performed on formalin-fixed, paraffin-embedded tissue sections. Briefly, tissue slides were subjected to heat-mediated antigen retrieval following deparaffinization and rehydration. Endogenous peroxidase activity was quenched by treatment with hydrogen peroxide. The sections were then incubated with the designated primary antibody overnight at 4 °C. Subsequent steps involved incubation with a biotinylated secondary antibody for 30 min, followed by signal amplification using a horseradish peroxidase-conjugated streptavidin complex. The chromogenic signal was developed using diaminobenzidine (DAB), after which the slides were counterstained, dehydrated, and mounted for microscopic imaging and analysis.

### TUNEL/Ki-67 assay in xenograft slides

Apoptosis and proliferation within tissue sections were visualized and quantified using TUNEL and Ki-67 fluorescence assays, respectively. The tissue slides were first deparaffinized, rehydrated, and then subjected to antigen retrieval by heating in a citric acid buffer at 95 °C for 8 min. After cooling and washing with PBS, non-specific binding sites were blocked via goat serum. The slides were then incubated with a reaction mixture containing fluorescently labeled TUNEL or Ki-67 (provided by Biyuntian, Wuxi, China), alongside a DAPI nuclear counterstain (Biyuntian). Following final wash steps, the sections were mounted and imaged using a ZEISS confocal laser scanning microscope to detect and quantify fluorescence signals.

### Statistical analysis

All quantitative data are presented as the mean ± standard deviation (SD) derived from five independent biological replicates for all in vitro experiments. The assumption of normality for all datasets was confirmed prior to statistical testing. For comparisons involving two distinct experimental groups, statistical significance was determined using a two-tailed, unpaired Student’s t-test. For analyses involving three or more groups, a one-way analysis of variance (ANOVA) was performed, followed by a Student-Newman-Keuls (SNK) post hoc test. A *P*-value of less than 0.05 was established as the criterion for statistical significance. All statistical computations were performed using standard statistical software packages (SPSS v.23.0).

#### Ethical approval

All methods were performed in accordance with the relevant guidelines and regulations. Human tissues and primary human cells were provided by the participants with written-informed consent. The management of these human tissue samples and cells followed protocols sanctioned by the Soochow University Ethics Committee (SUDA20230203A15), aligning with the Declaration of Helsinki standards. All animal procedures were approved by the Institutional Animal Care and Use Committee of Soochow University.

## Results

### Elevated *SNRPC* expression is a robust prognostic and diagnostic biomarker in glioma

**Fig. 1:**
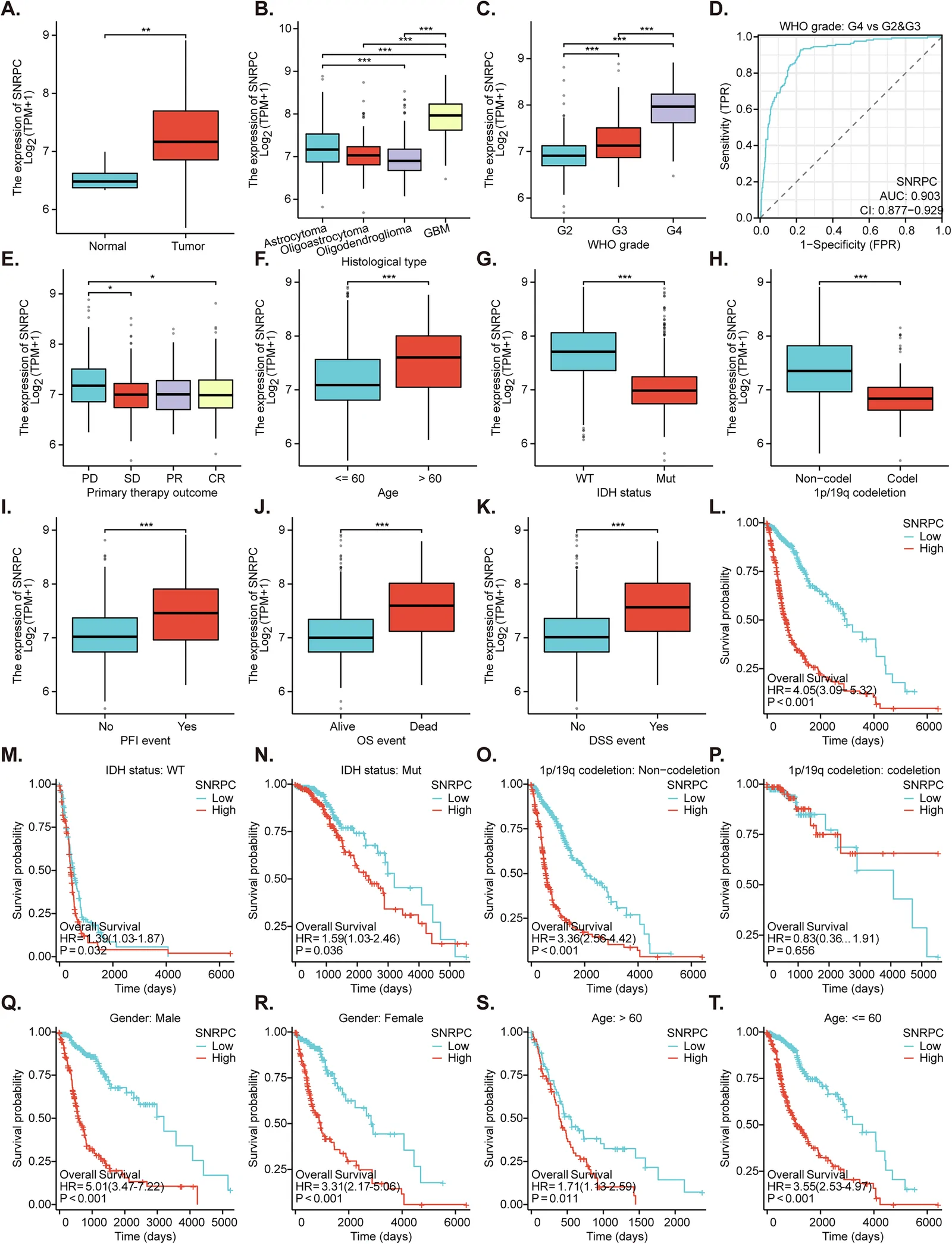
Elevated *SNRPC* expression is a robust prognostic and diagnostic biomarker in glioma. The expression of *SNRPC* in tumor versus normal brain tissues was analyzed using RNA sequencing data from the TCGA-GBMLGG cohort (**A**). The expression levels across different histological types were also compared (**B**). A comparison of *SNRPC* expression in glioma tissues across different WHO grades was performed (**C**), and its diagnostic value for distinguishing Grade IV from lower-grade gliomas was evaluated using ROC curve analysis (**D**). Expression levels were also correlated with primary therapy outcome (**E**), patient age (**F**), *IDH* mutation status (**G**), and 1p/19q codeletion status (**H**). Kaplan–Meier survival analysis was used to evaluate the correlation between *SNRPC* expression and progression-free interval (**I**), overall survival status (**J**), and disease-specific survival status (**K**). The prognostic significance of *SNRPC* was assessed for the entire TCGA-GBMLGG cohort (**L**) and within specific subgroups, including IDH wild-type (**M**) and mutant (**N**) gliomas, as well as 1p/19q non-codeleted (**O**) and codeleted (**P**) tumors. The prognostic value was further confirmed in subgroups based on gender (**Q** and **R**) and age (**S** and **T**). “TPM” stands for transcripts per million. “HR” stands for hazard ratio. Data were derived from the TCGA glioma dataset. *P < 0.05, **P < 0.01, ***P < 0.001.

A detailed analysis of RNA sequencing data from the comprehensive TCGA GBM and lower-grade glioma (LGG) cohort revealed a profound and statistically significant upregulation of *SNRPC* transcripts in tumor tissues relative to normal brain tissue (Fig. 1A). A more histological analysis confirmed this observation, showing that *SNRPC* expression was highest in the highly aggressive GBM tumors, with progressively lower expression levels in oligodendroglioma and astrocytoma (Fig. 1B). SNRPC expression increased in alignment with ascending WHO tumor grades, with Grade IV tumors showing the highest expression (Fig. 1C). This robust, grade-dependent expression pattern was validated by receiver operating characteristic (ROC) curve analysis, which demonstrated that *SNRPC* expression is a highly valuable biomarker for distinguishing Grade IV from LGGs, achieving an impressive area under the curve (AUC) of 0.903 (Fig. 1D).

The expression of *SNRPC* was also intricately linked to clinical outcomes and molecular characteristics. As depicted in Fig. 1E, patients who achieved a more favorable clinical response, such as stable disease (SD) or complete remission (CR), exhibited lower *SNRPC* expression compared to those with progressive disease (PD). This suggests a strong inverse correlation between *SNRPC* levels and positive treatment response. Consistent with its role in more aggressive disease, *SNRPC* expression was also found to be markedly higher in patients over 60 years of age (Fig. 1F). *SNRPC* expression was significantly lower in tumors with *an IDH* (*isocitrate dehydrogenase*) mutation (Fig. 1G) and in those with 1p/19q co-deletion (Fig. 1H), both of which are established molecular markers for a more favorable prognosis [10].

Further analysis confirmed a strong and consistent correlation between elevated *SNRPC* expression and a higher likelihood of negative clinical events. As shown in Fig. 1I, high *SNRPC* expression was significantly associated with a higher risk of experiencing a progression-free interval (PFI) event. Similarly, a strong link was observed between high *SNRPC* levels and patient mortality, with significantly higher expression in patients who were deceased from any cause (Fig. 1J) or specifically from their disease (Fig. 1K). These findings were further substantiated by Kaplan-Meier survival analysis, which consistently demonstrated that high expression of *SNRPC* was associated with significantly reduced overall survival for the entire TCGA-GBMLGG cohort (Fig. 1L). This prognostic value was maintained across crucial clinical subgroups, indicating that high *SNRPC* expression is a powerful and independent predictor of poorer survival outcomes in both *IDH* wild-type (Fig. 1M) and mutant tumors (Fig. 1N), as well as in tumors with 1p/19q non-codeletion (Fig. 1O) and codeletion (Fig. 1P). Finally, subgroup analyses confirmed the consistent prognostic significance of *SNRPC* expression regardless of gender (Fig. 1Q, R) and age (Fig. 1S, T), as high expression was associated with significantly reduced overall survival in both male and female patients, as well as in patients younger and older than 60 years of age. Collectively, these TCGA findings establish elevated *SNRPC* expression as a powerful, independent biomarker for the diagnosis, aggressive progression, and poor prognosis of glioma.

### Single-cell and functional analysis reveal high *SNRPC* expression in malignant glioma cells and its association with metabolic processes

**Fig. 2:**
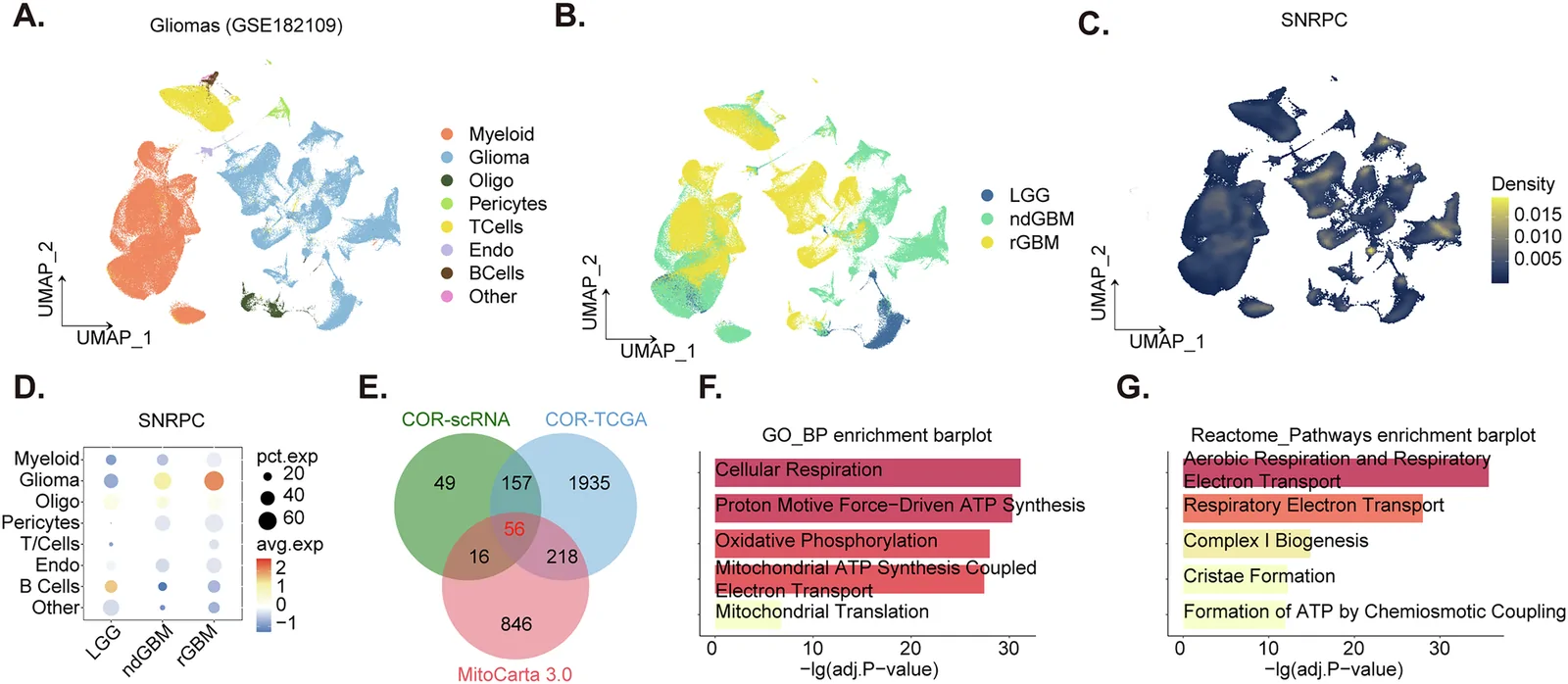
Single-cell and functional analysis reveal high *SNRPC* expression in malignant glioma cells and its association with metabolic processes. Single-cell RNA sequencing data (GSE182109) from glioma tissues were analyzed. Dimensionality reduction plots were generated to visualize distinct cell populations based on cell annotations (**A**) and clinical conditions (**B**). Expression density and dot plots were used to assess the expression of *SNRPC* across different cell types and clinical conditions (**C**, **D**). Co-expression analysis was performed to identify genes correlated with *SNRPC* using single-cell sequencing, TCGA-LGGGBM and MitoCarta 3.0data, and the overlap was visualized using a Venn diagram (**E**). The final set of co-expressed genes was then subjected to functional enrichment analysis using Gene Ontology (GO) (**F**) and Reactome pathway databases (**G**).

To understand the cellular context of *SNRPC* expression, single-cell RNA sequencing data from glioma tissues were analyzed using the GSE dataset (GSE182109). Dimensionality reduction plots, based on cell annotations provided by the original authors [31], visualized the distinct cell populations within the glioma microenvironment (Fig. 2A), while also highlighting the separation of three clinical conditions: LGG, newly diagnosed glioblastoma (ndGBM), and recurrent glioblastoma (rGBM) (Fig. 2B). Subsequent expression analysis revealed a striking concentration of *SNRPC* in the malignant glioma cell cluster. Both expression density plots (Fig. 2C) and corresponding dot plots (Fig. 2D) confirmed that *SNRPC* expression was highest in these cancer cells, with particularly elevated levels in the highly aggressive rGBM cells.

To investigate the molecular mechanisms underlying the role of *SNRPC*, a comprehensive co-expression analysis was performed. Genes correlated with *SNRPC* were identified from two distinct datasets: single-cell RNA sequencing (scRNA) and bulk TCGA-GBM data. This cross-referencing approach, visually represented by the Venn diagram in Fig. 2E, allowed us to robustly identify 213 shared co-expressed genes by using a strict filter of *P* < 0.05 and a Pearson’s correlation coefficient (*R*) greater than 0.5. To further refine the functional role of these genes, an intersection was performed with the MitoCarta 3.0 database, which revealed that 56 of the co-expressed genes were associated with mitochondrial function. Functional enrichment analysis of these 56 mitochondrial genes showed a strong and highly significant enrichment in core cellular processes (Fig. 2E). Both Gene Ontology (GO) and Reactome pathway analyses (Fig. 2F, G) indicated a primary role in fundamental cellular metabolism, with significant enrichment of terms such as cellular respiration, mitochondrial ATP electron transport chain, and oxidative phosphorylation (OXPHOS) (Fig. 2F, G). These results suggest that the elevated expression of *SNRPC* in glioma cells is functionally linked to a metabolic program centered on mitochondrial activity and energy production.

### SNRPC expression is upregulated in locally-resected glioma tissues and various glioma cells

**Fig. 3:**
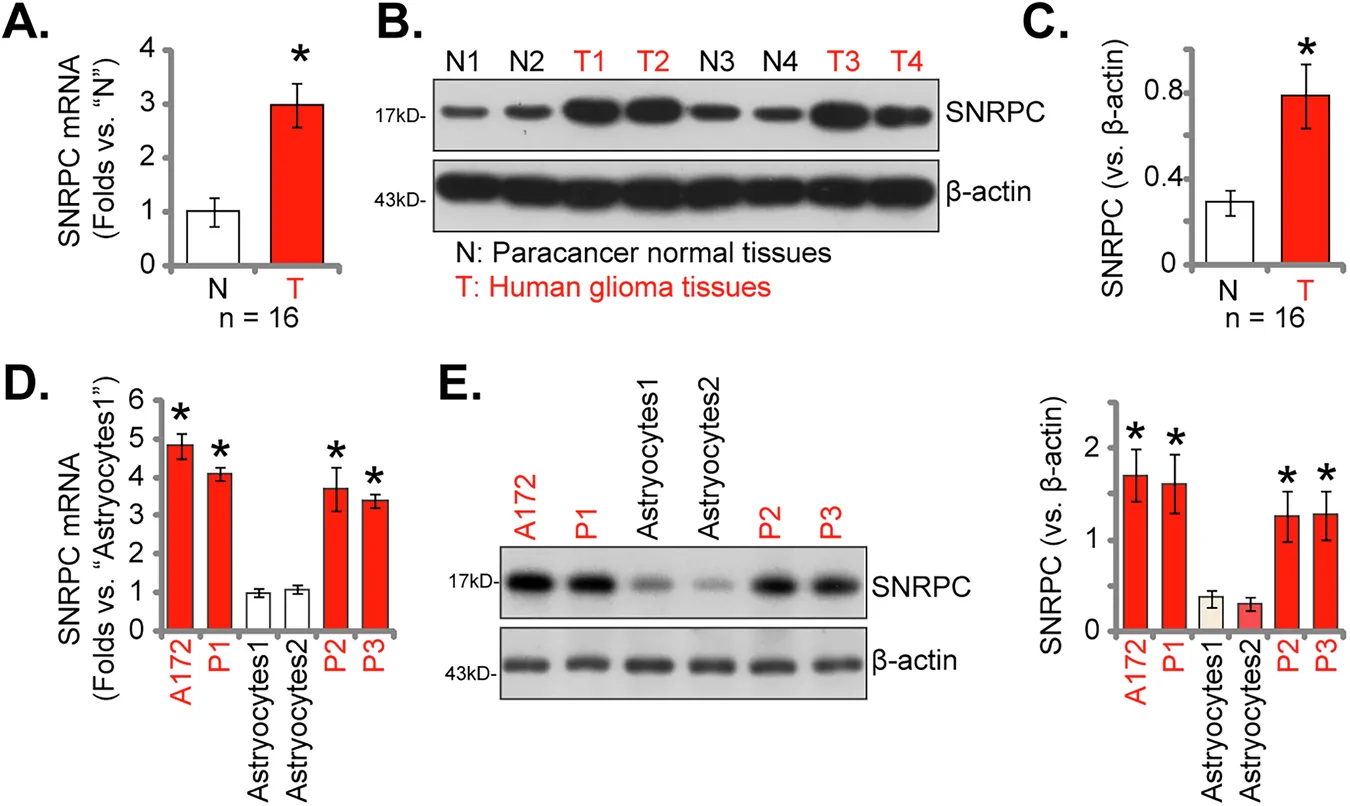
SNRPC expression is upregulated in locally resected glioma tissues and various glioma cells. Quantification of *SNRPC* mRNA levels in human glioma tissues (T, *n* = 16) compared to adjacent paracancerous normal tissues (N, *n* = 16) was determined by qRT-PCR (**A**). Representative Western blot analysis shows SNRPC protein expression in four glioma tumor samples (T1-T4) and adjacent paracancerous normal tissue samples (N1-N4), with the loading control (ß-actin) presented (**B**). Densitometric quantification of SNRPC protein levels from Western blot analysis was performed across the glioma tissue cohort (T, *n* = 16) and the normal tissue cohort (N, *n* = 16) (**C**). *SNRPC* mRNA expression was measured by qRT-PCR in the established A172 glioma cell line and three independent primary glioma cell cultures (P1, P2, P3) compared to two normal human astrocyte primary cultures (Astrocytes1, Astrocytes2) (**D**). Representative Western blot analysis confirms SNRPC protein expression in A172, primary glioma cells (P1, P2, P3), and normal human astrocytes (Astrocytes1, Astrocytes2), with loading control (ß-actin) shown (**E**). Results are shown as mean ± standard deviation (SD). Significance is denoted as * *P* < 0.05 versus the N tissues or Astrocytes1.

To further investigate the clinical relevance of SNRPC, its expression levels were analyzed in locally-resected human glioma tissues and various glioma cell types. As shown, *SNRPC* mRNA expression was found to be significantly upregulated in human glioma tissues (T, *n* = 16, reported previously [23, 29, 30]) compared to adjacent paracancerous normal tissues (N, *n* = 16) (Fig. 3A). The median expression of mRNA in glioma tissues was approximately 3-fold higher than in normal tissues (Fig. 3A). This finding was corroborated at the protein level. Western blot analysis revealed a clear elevation of SNRPC protein in representative tumor samples (T1-T4, from patient-1 to patient-4) compared to normal paracancerous samples (N1-N4) (Fig. 3B). Quantitative densitometric analysis of the Western blots confirmed a statistically significant increase in SNRPC protein levels in the glioma tissue cohort (T) compared to the normal tissue cohort (N) (Fig. 3C). Further analysis compared SNRPC expression in primary glioma cells and A172 established glioma cell line against normal human astrocytes. As shown in Fig. 3D, the *SNRPC* mRNA levels in the A172 glioma cell line and in three independent primary glioma cell cultures (P1, P2, and P3, reported previously [23, 29, 30]) were markedly higher than those observed in normal Astrocytes1 and Astrocytes2 (reported previously [23, 29, 30]). Similarly, the differential expression was confirmed by Western blot analysis of SNRPC protein (Fig. 3E). The glioma cell line (A172) and all three primary glioma cell types (P1, P2, and P3) exhibited significantly elevated SNRPC protein levels compared to the astrocyte controls, indicating that the heightened expression observed in whole tissue is maintained in the tumor cell population.

### SNRPC silencing attenuates malignant phenotypes across multiple glioma cell types

**Fig. 4:**
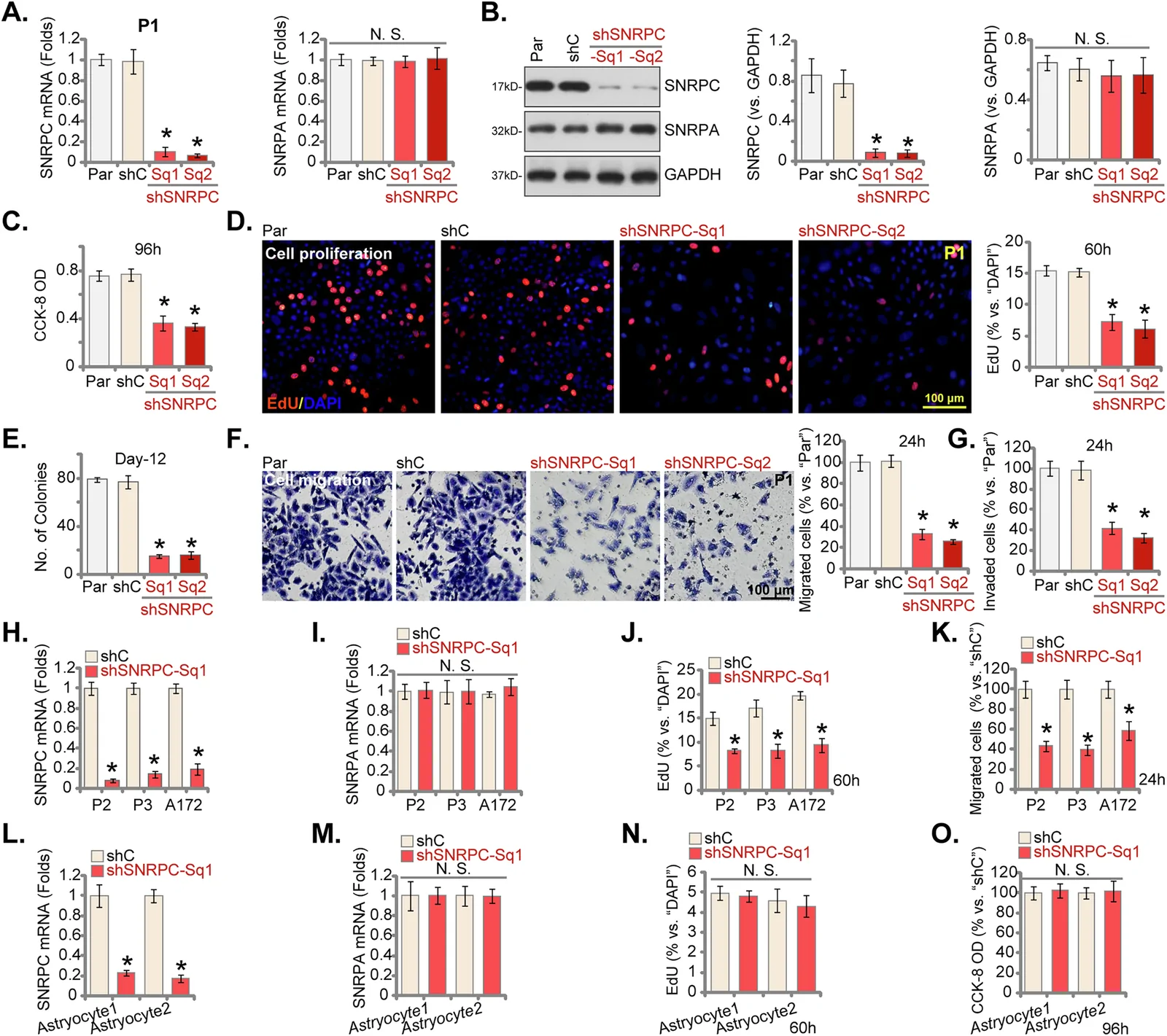
SNRPC silencing attenuates malignant phenotypes across multiple glioma cell types. *SNRPC* and *SNRPA* mRNA knockdown efficiency in P1 primary glioma cells with two short hairpin RNA constructs (shSNRPC-Sq1 and shSNRPC-Sq2) was evaluated by qRT-PCR against parental (Par) and scrambled control (shC) P1 glioma cells (**A**). Western blot analysis confirmed the knockdown of SNRPC protein in P1 glioma cells, with SNRPA protein assessed as a specificity control (**B**). Cell viability of SNRPC-silenced P1 glioma cells and control cells was assessed by the Cell Counting Kit-8 (CCK-8) assay at 96 h (**C**), and the proliferation rate at 60 h was determined by the EdU incorporation assay (**D**). Long-term survival was assessed using a colony formation assay, with colony counts quantified at Day-12 (**E**). Cell migration ability was quantified using a Transwell assay without Matrigel coating (**F**), and cell invasion potential was quantified using a Transwell assay with Matrigel coating (**G**). qRT-PCR analysis showed *SNRPC* and *SNRPA* mRNA levels in P2/P3 and A172 glioma cells following shSNRPC-Sq1 or shC transfection (**H**, **I**). Cells were cultured for designated hours, proliferation assessment via EdU incorporation assay was conducted (**J**), and cell migration was assessed using a Transwell assay (**K**). qRT-PCR analysis of *SNRPC* and *SNRPA* mRNA levels (**L**, **M**) was performed in normal human astrocytes (Astrocyte1, Astrocytes2) following shSNRPC-Sq1 or shC transfection. Cells were cultured for designated hours, proliferation assessment (**N**) and cell viability (**O**) were determined by EdU incorporation assay and CCK-8 assay, respectively. Results are shown as mean ± standard deviation (SD) from five independent biological replicates (*n* = 5). Significance is denoted as *P < 0.05 versus the shC control group. “NS” denotes non-significance. Scale bar, 100 μm.

To precisely define the functional role of the SNRPC, its expression was selectively silenced using two distinct short hairpin RNA (shRNA) constructs, shSNRPC-Sq1 and shSNRPC-Sq2 (targeting non-overlapping sequences), in P1 primary glioma cells. Figure 4A confirmed the successful transcriptional knockdown, showing a marked, significant reduction in *SNRPC* mRNA levels in the treated cells compared to both parental control (Par) and scrambled control (shC) P1 glioma cells. Expression of *SNRPA* mRNA levels was unchanged (Fig. 4A). This suppression was further validated at the protein level via Western blot analysis (Fig. 4B). Quantitation demonstrated that the shSNRPC P1 glioma cells exhibited an approximate 80–90% decrease in SNRPC protein expression, with no observable change in the expression of the related control protein, SNRPA (Fig. 4B). Functional assays demonstrated that SNRPC silencing severely compromised cell functions. The CCK-8 assay at 96 h revealed a substantial reduction of viability in both shSNRPC groups (Fig. 4C). This anti-proliferative effect was further quantified by the EdU incorporation assay (Fig. 4D), and the percentage of proliferating EdU-positive cells was significantly reduced in the shSNRPC-Sq1 and shSNRPC-Sq2 P1 glioma cells (Fig. 4D). Furthermore, cell colony formation (Fig. 4E) was profoundly inhibited, resulting in an approximate reduction in colony count by Day-12, indicating a critical role for SNRPC in maintaining tumor cell proliferation and survival. SNRPC depletion also significantly impaired the aggressive cellular phenotypes of migration and invasion, assessed using Transwell assays. The number of migrated cells (Fig. 4F) was decreased in both shSNRPC P1 glioma cells relative to the shC control. Similarly, the ability of cells to penetrate an extracellular matrix barrier was severely hampered; the count of invaded cells through the Matrigel layer (Fig. 4G) was also reduced by in the shSNRPC-Sq1 and shSNRPC-Sq2 P1 glioma cells. These results underscore the necessity of SNRPC for effective tumor cell motility and invasive potential.

To investigate the functional role of various other small nuclear ribonucleoprotein (SNRP) components in glioma, P1 primary glioma cells were transfected with 200 nM of specific siRNAs targeting SNRPA, SNRPB, SNRPC, SNRPD1, and SNRPE. The silencing protocol involved two rounds of transfection at 24-hour intervals for a total duration of 48 h. Although quantitative RT-PCR confirmed the efficient and specific knockdown of each target gene (Fig. S2A), the phenotypic consequences were highly selective. As demonstrated by the CCK-8 viability assay at 96 h (Fig. S2B) and the EdU proliferation assay at 60 h (Fig. S2C), only the silencing of SNRPC resulted in a significant reduction in cell viability and a marked inhibition of proliferative capacity. In contrast, silencing of the other SNRP family members—SNRPA, SNRPB, SNRPD1, and SNRPE—failed to induce any observable inhibitory effects on P1 glioma cell viability/proliferation compared to control siRNA group (Fig. S2B, C). These findings collectively demonstrate that SNRPC is uniquely essential for maintaining the viability and driving the proliferation of P1 glioma cells.

To generalize these findings, the core experiments were replicated in two additional primary glioma cell types, P2 and P3, as well as in the established A172 cells. Successful knockdown of *SNRPC* mRNA using shSNRPC-Sq1 was confirmed in P2/P3 and A172 cells (Fig. 4H), while *SNRPA* mRNA levels remained stable (Fig. 4I). Consistent with the findings in P1 glioma cells, SNRPC suppression significantly reduced EdU incorporation in P2/P3 and A172 glioma cells (Fig. 4J), confirming the anti-proliferative effect. The inhibitory effect on cell migration by shSNRPC-Sq1 was also validated in P2/P3 and A172 glioma cells (Fig. 4K). To determine if the function of SNRPC is specific to glioma cells, the effect of knockdown was tested in primary human astrocytes (Astrocyte1 and Astrocyte2 [23, 29, 30]). Although shSNRPC-Sq1 successfully lowered *SNRPC* mRNA levels (Fig. 4L) without affecting the control *SNRPA* mRNA (Fig. 4M), this reduction had no significant effect on normal cell proliferation, as determined by both EdU incorporation (Fig. 4N) and cell viability, as tested via the CCK-8 assay (Fig. 4O). This result indicates that the observed essential role of SNRPC is functionally restricted to the malignant glioma cells.

### SNRPC silencing induces apoptosis across multiple glioma cell types

**Fig. 5:**
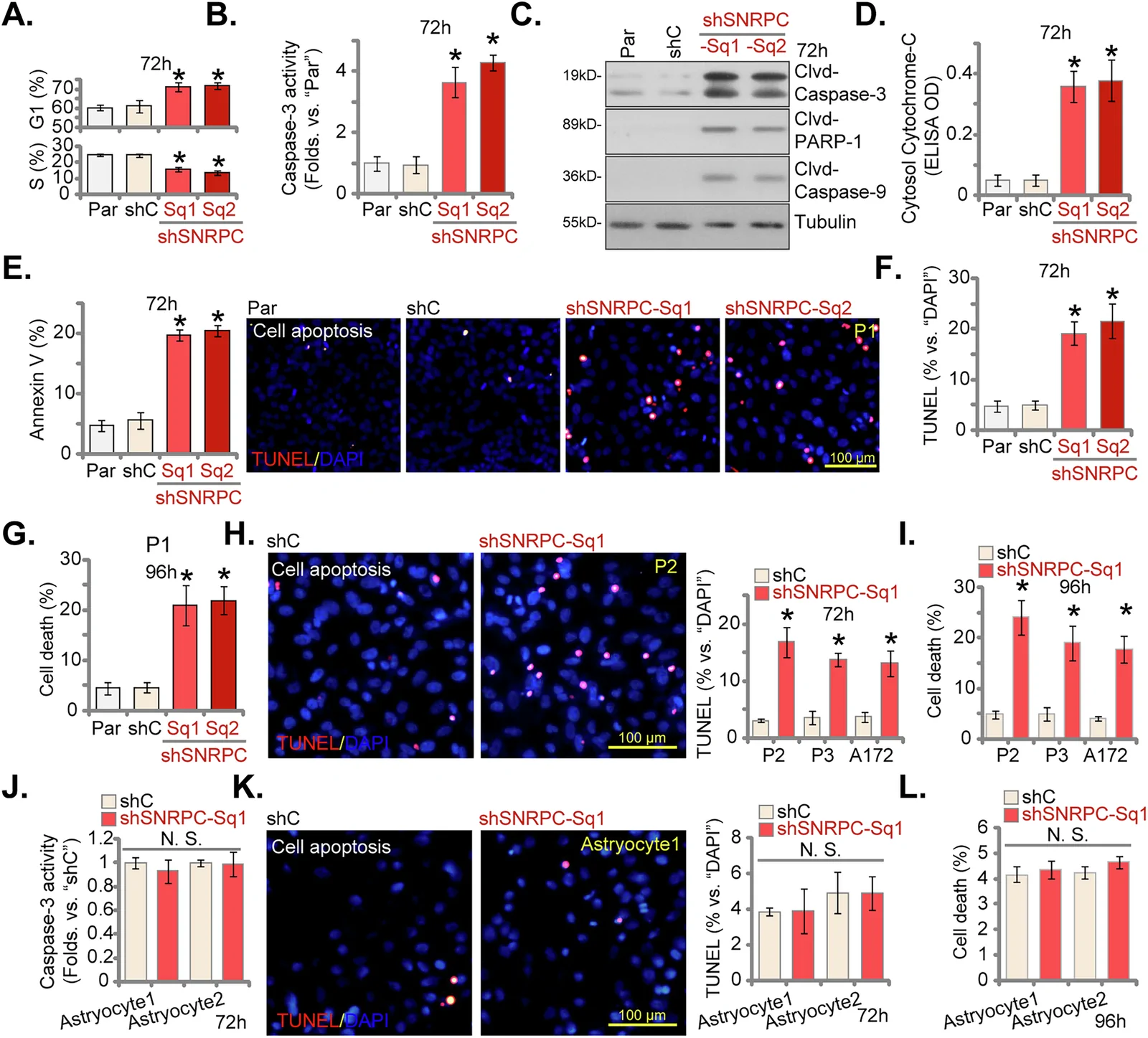
SNRPC silencing induces apoptosis across multiple glioma cell types. The cell cycle distribution of SNRPC-silenced, parental (Par) and scrambled shRNA control (shC) P1 glioma cells was determined by flow cytometry following propidium Iodide (PI) staining (**A**), with caspase-3 activity quantified at 72 h (**B**), and Western blot analysis confirming apoptotic induction by detecting cleaved Caspase-3, Caspase-9 and cleaved Poly(ADP-ribose) polymerase-1 (PARP-1) (**C**). Cytochrome C release from the mitochondria into the cytosol in above P1 glioma cells was assessed via an ELISA assay (**D**). Total apoptotic cells (Annexin V positive) were quantified by Annexin V/PI staining (**E**), with DNA fragmentation quantified using TUNEL staining (**F**). Cell death in above P1 glioma cells was quantified via Trypan blue staining (**G**). The apoptosis was quantified via nuclear TUNEL staining (**H**) and cell death was measured via Trypan blue staining (**I**) in shC control or SNRPC-silenced (via shSNRPC-Sq1) P2/P3 primary glioma cells and A172 cells. The normal human astrocytes (Astrocyte1, Astrocytes2) with shSNRPC-Sq1 or shC transfection were cultivated for designated hours, with Caspase-3 activity measured via an ELISA assay (**J**), apoptosis quantified by TUNEL staining (**K**), and cell death quantified by Trypan blue staining (**L**) measured. Results are shown as mean ± standard deviation (SD) from five independent biological replicates (*n* = 5). Significance is denoted as *P < 0.05 versus the shC control group. “NS” denotes non-significance. Scale bar, 100 μm.

Mechanistic investigation revealed that the anti-proliferative effect of SNRPC silencing (see Fig. 4) is mediated through the induction of programmed cell death and a perturbation of cell cycle kinetics in glioma cells. Flow cytometric analysis (Fig. 5A) demonstrated a significant reduction in S-phase cells at 72 h in shSNRPC-Sq1 and shSNRPC-Sq2 P1 glioma cells, accompanied by an increase in G1-phase cells, indicating G1–S progression arrest (Fig. 5A). This kinetic alteration coincided with a robust induction of apoptosis. At 72 h, Caspase-3 activity, a central executioner of apoptosis, was significantly elevated in the shSNRPC P1 glioma cells compared to controls (Fig. 5B). Western blot analysis (Fig. 5C) provided biochemical confirmation, showing increased cleavages of both Caspase-3, Caspase-9 and Poly(ADP-ribose) polymerase (PARP-1) in SNRPC-silenced P1 glioma cells. Furthermore, the induction of the intrinsic pathway was demonstrated by a significant enhancement of Cytochrome C release from the mitochondria into the cytosol (Fig. 5D). Annexin V/Propidium Iodide (PI) staining (Fig. 5E) showed that the total population of apoptotic cells (Annexin V positive) increased significantly in P1 glioma cells with SNRPC silencing. Further quantification revealed an increase in TUNEL-positive P1 glioma cells at 72 hours following SNRPC shRNA treatment, verifying increased DNA fragmentation associated with apoptosis (Fig. 5F). Consistent with these findings, the overall percentage of dead cells measured by the Trypan blue staining assay (Fig. 5G) was significantly increased at 96 hours in P1 glioma cells with SNRPC silencing.

The pro-apoptotic effect was validated across other glioma cells as well, including P2/P3 primary cells and A172 immortalized cells. In these glioma cells, SNRPC-Sq1 treatment significantly enhanced apoptosis (tested via nuclear TUNEL staining, Fig. 5H). Moreover, a significant increase in cell death, measured via Trypan blue staining, was detected in SNRPC-silenced P2/P3 and A172 glioma cells (Fig. 5I). Crucially, SNRPC knockdown in normal human astrocytes (Astrocyte1 and Astrocyte2) did not induce apoptosis. Neither Caspase-3 activity (Fig. 5J) nor the percentage of TUNEL-positive cells (Fig. 5K) showed any significant change. SNRPC-Sq1-induced silencing of SNRPC also failed to induced significant cell death in the astrocytes (Fig. 5L), as tested via the Trypan blue staining assays. In primary murine cortical neurons, transduction with murine SNRPC shRNA (shmSNRPC) markedly reduced SNRPC protein levels without altering SNRPA expression (Fig. S3A). Notably, SNRPC silencing did not significantly affect neuronal viability (CCK-8 OD; Fig. S3B), cell death (Trypan blue staining assay; Fig. S3C), or neuronal morphology (MAP2 staining, Fig. S3D). This finding reinforces the conclusion that SNRPC is selectively required for the survival and malignant phenotype of the malignant glioma cells.

### SNRPC silencing induces metabolic and mitochondrial dysfunction in glioma cells

Emerging evidence now indicates that mitochondrial hyperfunction is a key metabolic hallmark of glioma, pivotal for its aggressive growth and progression [32, 33]. This state of heightened mitochondrial activity supplies the bioenergetic and biosynthetic precursors required to fuel relentless cell proliferation, invasion, and therapeutic resistance [32, 33]. This distinct metabolic phenotype exposes a potential therapeutic vulnerability, suggesting that targeting the pathways sustaining mitochondrial hyperfunction could constitute a novel and effective anti-glioma strategy.

**Fig. 6:**
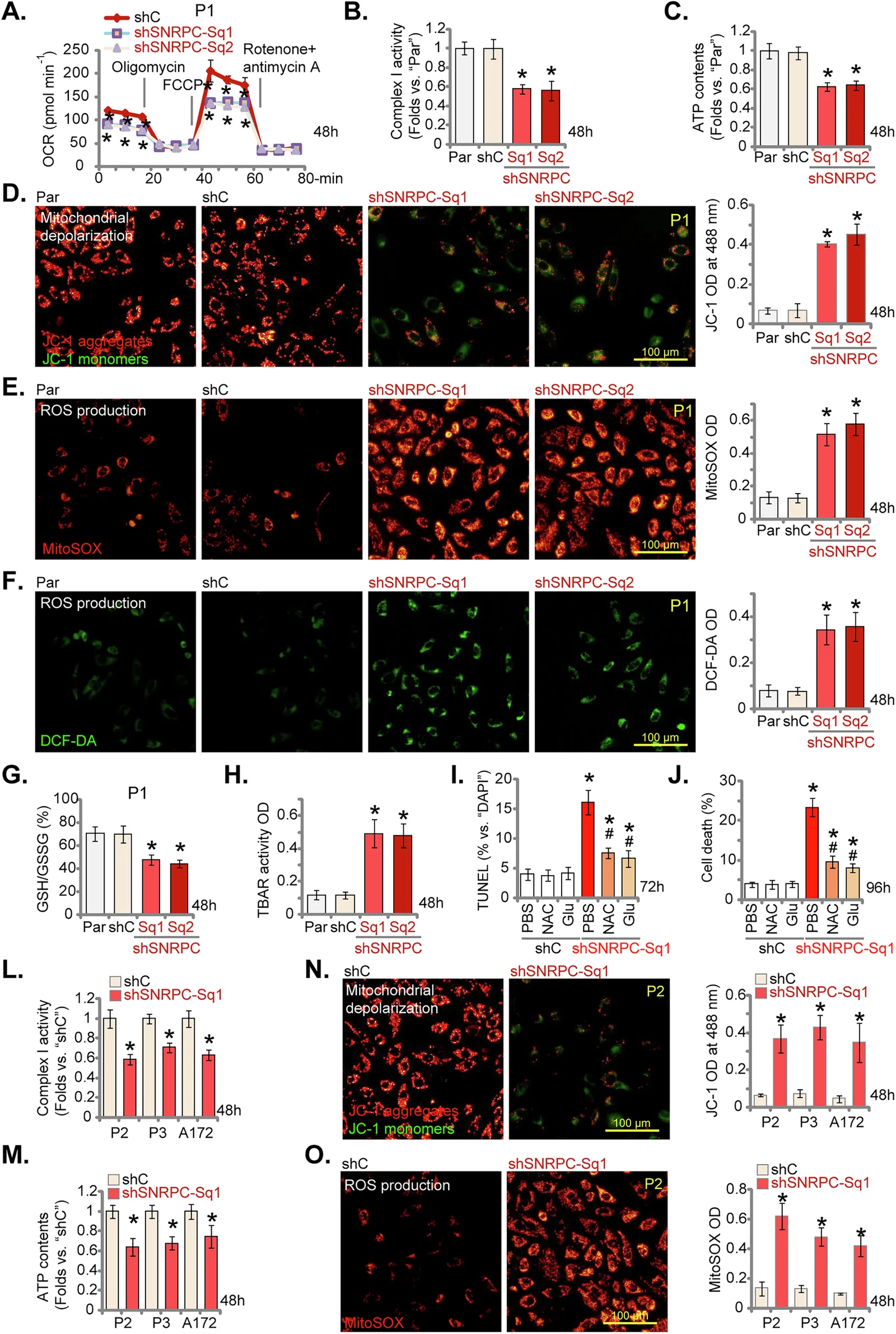
SNRPC silencing induces metabolic and mitochondrial dysfunction in glioma cells. Mitochondrial function in shSNRPC-Sq1, shSNRPC-Sq2 P1 glioma cells, parental (Par) control and scrambled shRNA control (shC) P1 glioma cells was evaluated by measuring oxygen consumption rate (OCR) using a Seahorse flux analyzer (**A**). The activity of mitochondrial electron transport chain (ETC) Complex I was measured (**B**), and cellular ATP content was quantified (**C**). Mitochondrial membrane potential was assessed via JC-1 staining (**D**), and mitochondrial reactive oxygen species (ROS) production was quantified using MitoSOX fluorescence (**E**). Total cellular ROS was measured using DCF-DA fluorescence (**F**), the cellular antioxidant status was quantified by measuring the reduced to oxidized glutathione ratio (GSH/GSSG%) (**G**), with lipid peroxidation assessed by measuring Thiobarbituric Acid Reactive Substances (TBAR) activity (**H**). The effect of glucose supplementation (10 mM) and the antioxidant N-acetylcysteine (NAC, 0.5 mM) on shSNRPC-Sq1-induced apoptosis in P1 glioma cells was assessed using the TUNEL assay (**I**) and on cell death using the Trypan blue exclusion assay (**J**). The activity of ETC Complex I (**L**), total cellular ATP content (**M**), mitochondrial membrane potential assessed via JC-1 staining (**N**), and mitochondrial ROS production quantified using MitoSOX fluorescence (**O**) were subsequently measured in SNRPC-silenced P2/P3 and A172 glioma cells and shC control cells. Results are shown as mean ± standard deviation (SD) from five independent biological replicates (*n* = 5). Significance is denoted as *P < 0.05 versus the shC control group. For (**I**, **J**), ^#^ *P* < 0.05 indicates significance compared to PBS treatment. “NS” denotes non-significance. Scale bar, 100 μm.

Consistent with the bioinformatic predictions linking SNRPC to metabolic and mitochondrial activity, its functional necessity for mitochondrial respiration was experimentally confirmed using the Seahorse flux analyzer. Figure 6A demonstrated that SNRPC knockdown in P1 glioma cells profoundly compromised key parameters of mitochondrial function. Both shSNRPC-Sq1 and shSNRPC-Sq2 cells exhibited a significant decrease in the basal oxygen consumption rate (OCR) compared to the shC control. A substantial reduction in maximal respiration (FCCP-induced OCR) further indicated a fundamental defect in the capacity and efficiency of the electron transport chain (ETC) (Fig. 6A). This respiratory defect was also accompanied with reduced the activity of mitochondrial ETC Complex I (Fig. 6B). Functionally, this bioenergetic failure resulted in a depletion of cellular energy reserves, with ATP content reduced in the SNRPC-silenced P1 glioma cells (Fig. 6C). The metabolic collapse was physically evidenced by immediate mitochondrial damage. Assessment of mitochondrial membrane potential via JC-1 staining (Fig. 6D) revealed a pronounced shift toward the monomeric (green) form, signifying widespread mitochondrial depolarization and loss of potential in shSNRPC P1 glioma cells (Fig. 6D). This mitochondrial dysfunction subsequently triggered substantial oxidative stress. Both shSNRPC P1 glioma cells exhibited significantly elevated mitochondrial reactive oxygen species (ROS) production, confirmed by increased MitoSOX fluorescence intensity (Fig. 6E). Correspondingly, total cellular ROS also increased, measured by DCF-DA fluorescence (Fig. 6F). The cellular defense system was simultaneously overwhelmed: the ratio of reduced to oxidized glutathione (GSH/GSSG%), a key measure of antioxidant reserve, was significantly decreased (Fig. 6G). The downstream consequence of this uncontrolled oxidative damage was lipid peroxidation, indicated by a significant increase in TBAR activity (Fig. 6H).

Next, P1 glioma cells were supplemented with glucose or treated with the antioxidant N-acetylcysteine (NAC). Both treatments effectively reversed the cytotoxic phenotype by SNRPC silencing. TUNEL assay (Fig. 6I) and trypan blue exclusion assay (Fig. 6J) indicated that NAC or glucose supplementation significantly mitigated apoptosis and death caused by shSNRPC-Sq1 in P1 glioma cells. These findings indicate that metabolic dysfunction and ROS production constitute essential, causal components of the apoptotic pathway activated by SNRPC silencing in glioma cells. The effect of SNRPC silencing on mitochondrial integrity was confirmed in the P2/P3 glioma cells and A172 cell line. Consistent with the P1 cells’ findings, shSNRPC-Sq1 treatment significantly reduced ETC Complex I activity (Fig. 6L) and caused a decline in total ATP content (Fig. 6M) across P2/P3 and A172 glioma cells. Furthermore, these glioma cells also displayed robust mitochondrial depolarization (Fig. 6N) and increased mitochondrial ROS production (MitoSOX fluorescence intensity increasing, Fig. 6O), confirming that the profound metabolic and mitochondrial dysfunction is a conserved consequence of SNRPC silencing in multiple malignant glioma cells.

### SNRPC knockout by CRISPR/Cas9 impairs mitochondrial function, bioenergetics, and malignant phenotypes in glioma cells

**Fig. 7:**
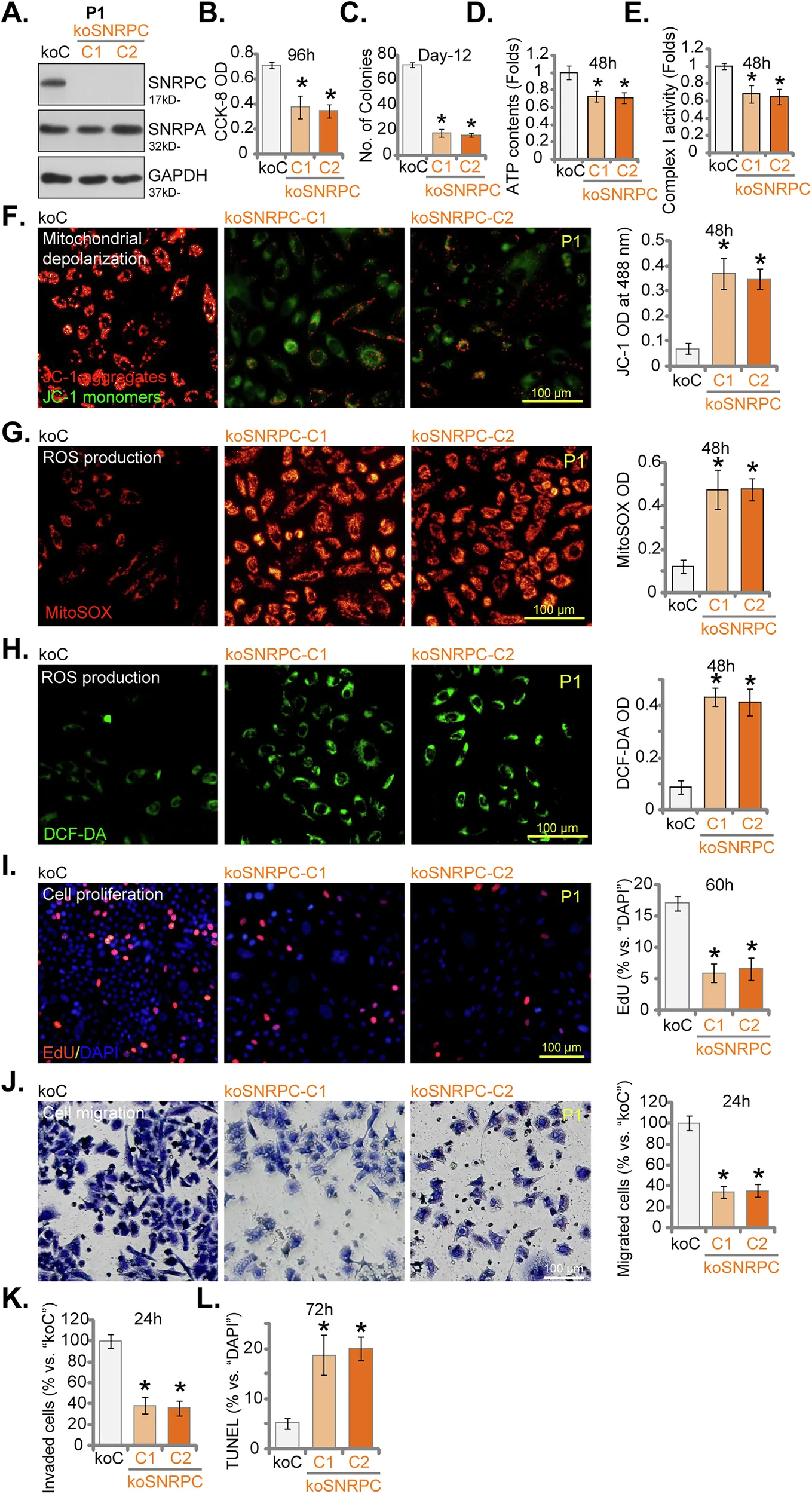
SNRPC knockout by CRISPR/Cas9 impairs mitochondrial function, bioenergetics, and malignant phenotypes in glioma cells. Western blot analysis verified the genetic ablation of SNRPC and confirmed SNRPA expression in two CRISPR/Cas9-SNRPC-KO P1 glioma clones (koSNRPC-C1 and koSNRPC-C2) compared to the knockout control cells (koC) (**A**). The above cells were cultivated for indicated time periods, cell viability was measured using the CCK-8 assay (**B**), and long-term survival was assessed by quantifying viable cell colonies (**C**). Cellular ATP content (**D**) and mitochondrial ETC Complex I activity (**E**) were quantified. Mitochondrial membrane potential was assessed using JC-1 fluorescence (**F**), mitochondrial ROS production was quantified using MitoSOX fluorescence (**G**), and total cellular ROS was measured using DCF-DA fluorescence (**H**). Proliferation rate was determined by the EdU incorporation assay (**I**), and cell mobility phenotypes were assessed by quantifying cell migration (**J**) and cell invasion (**K**) using Transwell assays. Apoptosis was assessed by quantifying DNA fragmentation using the TUNEL staining assay (**L**). Results are shown as mean ± standard deviation (SD) from five independent biological replicates (*n* = 5). Significance is denoted as *P < 0.05 versus the koC control group. “NS” denotes non-significance. Scale bar, 100 μm.

To provide definitive genetic confirmation of SNRPC’s role, the gene was ablated in P1 glioma cells using a CRISPR/Cas9-SNRPC-KO construct, yielding two stable knockout clones, koSNRPC-C1 and koSNRPC-C2. Western blot analysis (Fig. 7A) verified successful protein depletion, showing negligible SNRPC expression in both koSNRPC-C1 and koSNRPC-C2 P1 glioma cells compared to the knockout control cells (koC), while the expression of SNRPA remained stable (Fig. 7A). Both clones exhibited a significant reduction in overall cell viability, as measured by the CCK-8 assay at 96 h (Fig. 7B). In addition, CRISPR/Cas9-induced SNRPC KO decreased the number of viable P1 glioma cell colonies (Fig. 7C). Consistent with the shRNA knockdown results (Fig. 6), SNRPC KO resulted in profound metabolic and mitochondrial dysfunction. This bioenergetic defect was evidenced by a substantial decrease in ATP content (Fig. 7C) and a corresponding reduction in ETC Complex I activity (Fig. 7D) at 48 h. The mitochondrial membrane potential was severely compromised, indicated by a significant increase in the monomeric (green) form of JC-1 fluorescence (Fig. 7F). It also led to a marked increase in oxidative stress. Both mitochondrial ROS production (MitoSOX, Fig. 7G) and total cellular ROS (DCF-DA OD, Fig. 7H) were significantly elevated in the koSNRPC-C1 and koSNRPC-C2 P1 glioma cell clones compared to koC control cells. The genetic ablation of SNRPC reproduced the inhibitory effects on malignant phenotypes previously observed with shRNA-mediated silencing. The EdU incorporation assay showed a significant reduction in the percentage of proliferating cells (EdU-positive) in both koSNRPC lines (Fig. 7I). The koSNRPC P1 glioma cells exhibited a substantial decrease in both the number of migrated cells (Fig. 7J) and invaded cells (Fig. 7K). SNRPC knockout culminated in apoptosis induction. TUNEL staining apoptosis assay revealed a significant increase in DNA fragmentation in both koSNRPC P1 glioma cell clones (Fig. 7L). Thus, the use of CRISPR/Cas9 knockout method confirmed that SNRPC is essential for maintaining mitochondrial function, bioenergetic balance, and the malignant phenotypes of glioma cells.

### SNRPC overexpression enhances mitochondrial function, bioenergetics, and malignant phenotypes in glioma cells

**Fig. 8:**
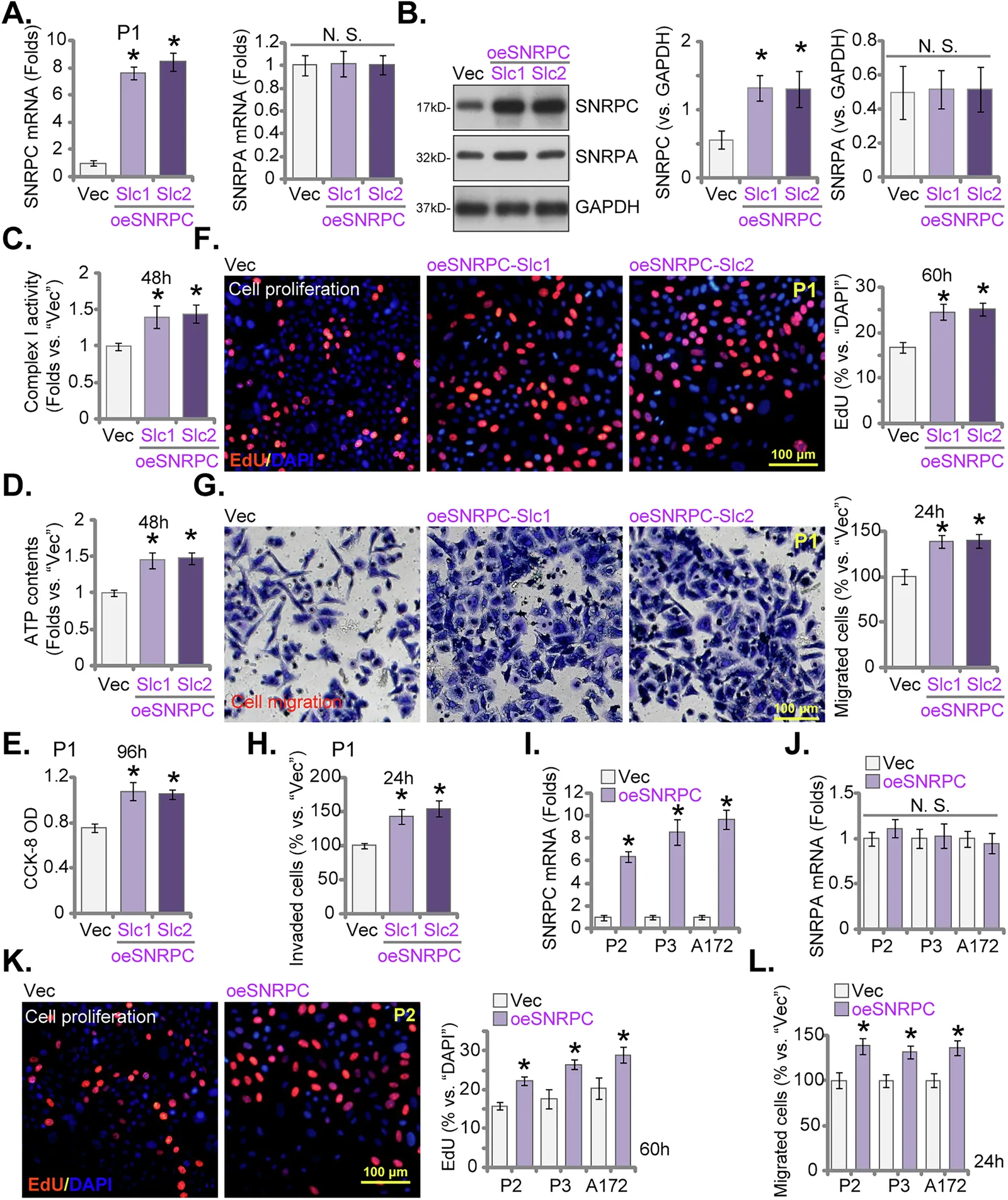
SNRPC overexpression enhances mitochondrial function, bioenergetics, and malignant phenotypes in glioma cells. qRT-PCR and Western blot assays confirmed *SNRPC* mRNA and protein overexpression in two stable P1 glioma cell clones (oeSNRPC-Slc1 and oeSNRPC-Slc2) with the lentiviral SNRPC overexpression construct, compared to the vector control (Vec), with *SNRPA* mRNA and protein assessed as a control (A and **B**). Cells were cultivated for designated hours, mitochondrial ETC Complex I activity (**C**) and cellular ATP content (**D**) were measured. Cell viability was measured using the CCK-8 assay (**E**), and proliferation rate was determined by the EdU incorporation assay (**F**). Cell migration ability (**G**) and cell invasion potential (**H**) were quantified using Transwell assays. *SNRPC* mRNA overexpression efficiency was validated by qRT-PCR in P2, P3, and A172 cells with the lentiviral overexpression construct (**I**), with *SNRPA* mRNA assessed as a control (**J**). Proliferation was assessed via the EdU incorporation assay in these glioma cells (**K**), and cell migration was assessed using a Transwell assay (**L**). Results are shown as mean ± standard deviation (SD) from five independent biological replicates (*n* = 5). Significance is denoted as *P < 0.05 versus the Vec control group. “NS” denotes non-significance. Scale bar, 100 μm.

To complement the shRNA/KO studies, SNRPC was constitutively overexpressed (oeSNRPC) in P1 glioma cells using a lentiviral overexpression construct which successfully yielded two stable cell clones following puromycin selection, namely oeSNRPC-Slc1 and oeSNRPC-Slc2. Figure 8A confirms successful overexpression, showing a significant increase in *SNRPC* mRNA levels compared to the vector control (“Vec”), without affecting *SNRPA* mRNA levels (Fig. 8A). This increase was also validated at the protein level by Western blot analysis (Fig. 8B), and again SNRPA protein levels were unchanged (Fig. 8B). The elevated SNRPC expression resulted in a corresponding augmentation of mitochondrial and bioenergetic function in P1 glioma cells. Both overexpression colonies exhibited significant increases in ETC Complex I activity (Fig. 8C) and ATP contents (Fig. 8D). Functionally, this led to a significant enhancement of CCK-8 OD (Fig. 8E). The EdU incorporation assay showed an increase in the percentage of proliferating (EdU-positive) cells in both overexpression P1 glioma cell colonies (Fig. 8F). The ability of cells to migrate was significantly enhanced, with the number of migrated cells increased (Fig. 8G). Similarly, the invasive potential of P1 glioma cells was boosted after SNRPC overexpression (Fig. 8H).

These pro-cancer effects were confirmed in other glioma cells. *SNRPC* mRNA overexpression (oeSNRPC) was successfully validated in P2, P3, and A172 cells using the same lentiviral construct (Fig. 8I), with *SNRPA* mRNA levels remaining unchanged (Fig. 8J). Consistent with the findings in P1 glioma cells, SNRPC overexpression significantly enhanced proliferation (Fig. 8K), as evidenced by increased EdU incorporation across P2, P3, and A172 cells. Furthermore, cell migration was augmented in all three glioma cell types (Fig. 8L). These results confirm that elevated SNRPC expression is functionally sufficient to drive enhanced mitochondrial metabolism and augmented malignant phenotypes across multiple glioma cell types.

### SNRPC regulates glioma malignancy via the TNFAIP2 signaling axis

**Fig. 9:**
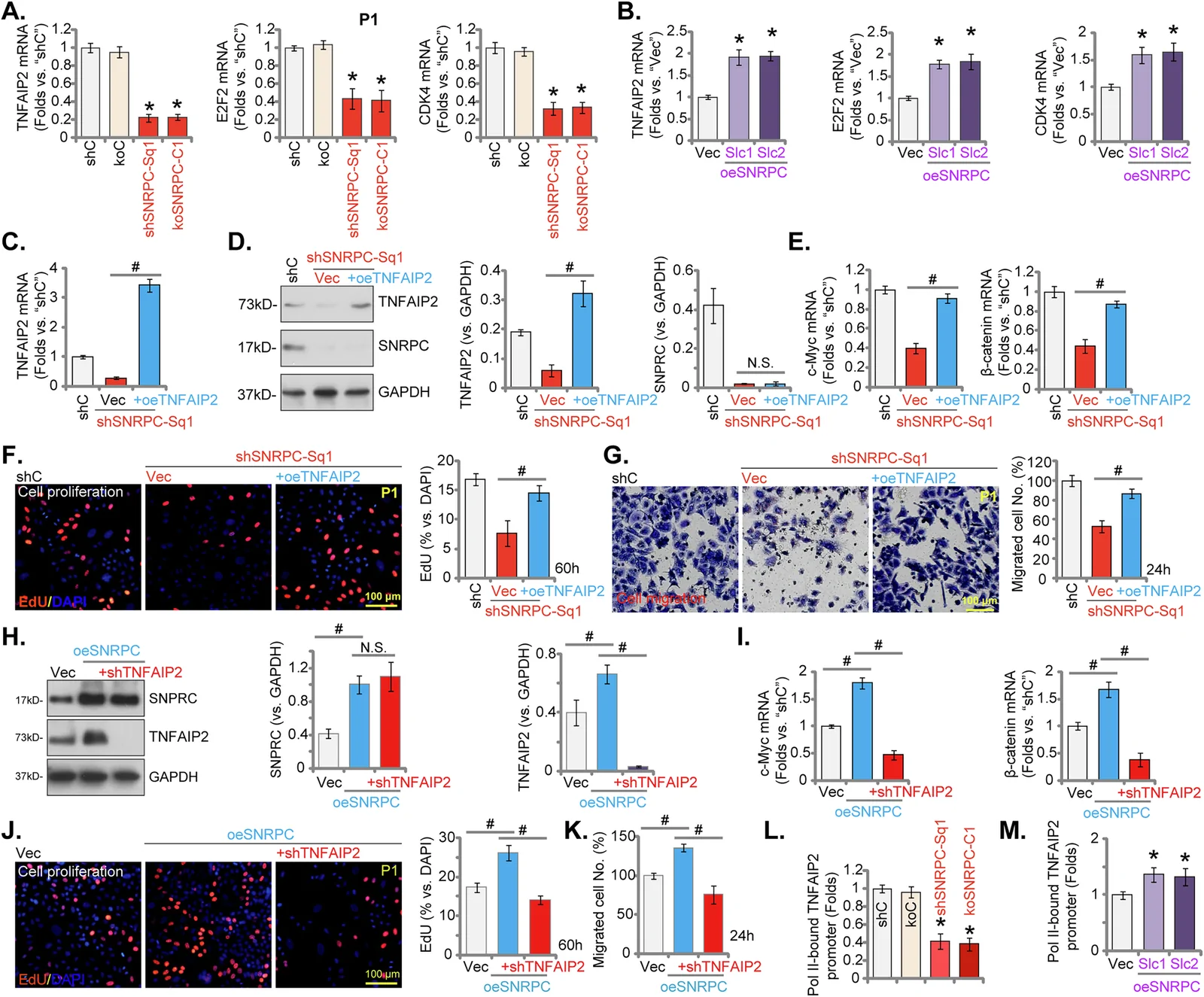
SNRPC regulates glioma malignancy via the TNFAIP2 signaling axis. qRT-PCR analysis of *TNFAIP2*, *E2F2*, and *CDK4* mRNA levels in P1 glioma cells. Expression was measured following SNRPC depletion using shRNA (shSNRPC-Sq1) or CRISPR/Cas9 knockout (koSNRPC-C1) (**A**), or following ectopic overexpression of SNRPC (oeSNRPC; clones Slc1 and Slc2) (**B**), as well as in their relative control P1 glioma cells (**A**, **B**). Rescue experiments were conducted in SNRPC-silenced P1 cells (shSNRPC-Sq1) by reintroducing a lentiviral TNFAIP2 overexpression construct (oeTNFAIP2). qRT-PCR and Western blotting were utilized to test expression of listed mRNAs and proteins (**C**–**E**). Cells were cultivated for indicated time periods, and cell proliferation was assessed using an EdU incorporation assay (**F**). Cell migration was evaluated using Transwell assays (**G**). The SNRPC-overexpressing P1 cells (oeSNRPC) were further transduced with or without the lentiviral*TNFAIP2* shRNA (shTNFAIP2), stable cells were formed. qRT-PCR and Western blotting were utilized to test expression of listed mRNAs and proteins (**H**, **I**). Cells were cultivated for indicated time periods, and cell proliferation was assessed using an EdU incorporation assay (**J**). Cell migration was evaluated using Transwell assays (**K**). ChIP-qPCR analysis of Pol II enrichment at the *TNFAIP2* promoter in P1 glioma cells with SNRPC shRNA (shSNRPC-Sq1) or CRISPR/Cas9 knockout (koSNRPC-C1) (**L**). ChIP-qPCR analysis of Pol II enrichment at the *TNFAIP2* promoter in P1 glioma cells overexpressing SNRPC or with empty vector (**M**). Results are shown as mean ± standard deviation (SD) from five independent biological replicates (*n* = 5). Significance is denoted as *P < 0.05 versus the shC/Vec control cell group. ^#^*P* < 0.05 (**C**–**K**). “NS” denotes non-significance. Scale bar, 100 μm.

Previous research has established that SNRPC is critical for maintaining U1 snRNP stability and regulating RNA Polymerase II-dependent transcriptional programs [20]. In triple-negative breast cancer (TNBC) cells, SNRPC knockdown markedly attenuated RNA Pol II binding and significantly downregulated the expression of key oncogenes, including tumor necrosis factor alpha-induced protein 2 (TNFAIP2), E2F transcription factor 2 (E2F2), and cyclin-dependent kinase 4 (CDK4) [20]. To determine if this regulatory mechanism is conserved in glioma, we modulated SNRPC levels in P1 glioma cells. Consistent with prior findings, depletion of SNRPC (via shRNA or CRISPR/Cas9 knockout) significantly downregulated the mRNA levels of RNA Polymerase II-dependent *TNFAIP2*, *CDK4*, and *E2F2* (Fig. 9A). Conversely, ectopic overexpression of SNRPC resulted in a marked upregulation of these transcripts (Fig. 9B).

Next, we investigated whether TNFAIP2 acts as a key functional downstream effector of SNRPC in glioma. To test this, we performed rescue assays by reintroducing TNFAIP2 into SNRPC-silenced P1 cells. Overexpression of TNFAIP2 using a lentiviral construct (oeTNFAIP2) successfully restored *TNFAIP2* mRNA and protein levels (Fig. 9C, D) in P1 glioma cells and rescued the expression of the downstream signaling targets *c-Myc* and *ß-catenin* [20] (Fig. 9E). Functionally, TNFAIP2 restoration partially reversed the suppression of cell proliferation (Fig. 9F) and migration (Fig. 9G) induced by SNRPC knockdown, confirming that SNRPC promotes glioma malignancy, at least in part, through TNFAIP2 modulation.

To further validate this regulatory hierarchy, we silenced TNFAIP2 in SNRPC-overexpressing (oeSNRPC) P1 cells. Transduction with a lentiviral TNFAIP2 shRNA (shTNFAIP2) effectively abolished the SNRPC-induced upregulation of TNFAIP2 protein without altering SNRPC expression levels in P1 glioma cells (Fig. 9H). Furthermore, TNFAIP2 silencing nullified the SNRPC-mediated upregulation of *c-Myc* and *ß-catenin* transcripts in P1 glioma cells (Fig. 9I). In functional assays, the enhanced cell proliferation (Fig. 9J) and migration (Fig. 9K) observed in SNRPC-overexpressing P1 cells were significantly attenuated by shTNFAIP2 treatment. In TNBC cells, SNRPC stabilizes the U1 snRNP complex, which acts as a transcriptional scaffold to facilitate the recruitment and enrichment of RNA Polymerase II (Pol II) specifically at the *TNFAIP2* promoter, thereby driving its over-expression [20]. Here, chromatin immunoprecipitation (ChIP) analysis demonstrated that the occupancy of Pol II at the *TNFAIP2* promoter was significantly attenuated upon SNRPC silencing or knockout in P1 glioma cells (Fig. 9L). In contrast, SNRPC overexpression promoted the recruitment of Pol II to the *TNFAIP2* promoter (Fig. 9M), suggesting that SNRPC is essential for the transcriptional activation of *TNFAIP2* also in primary human glioma cells. These data indicate that the SNRPC-TNFAIP2 axis is pivotal for regulating oncogenic signaling and malignant behaviors in glioma cells.

### SNRPC knockdown suppresses subcutaneous glioma xenograft growth and disrupts mitochondrial bioenergetics

**Fig. 10:**
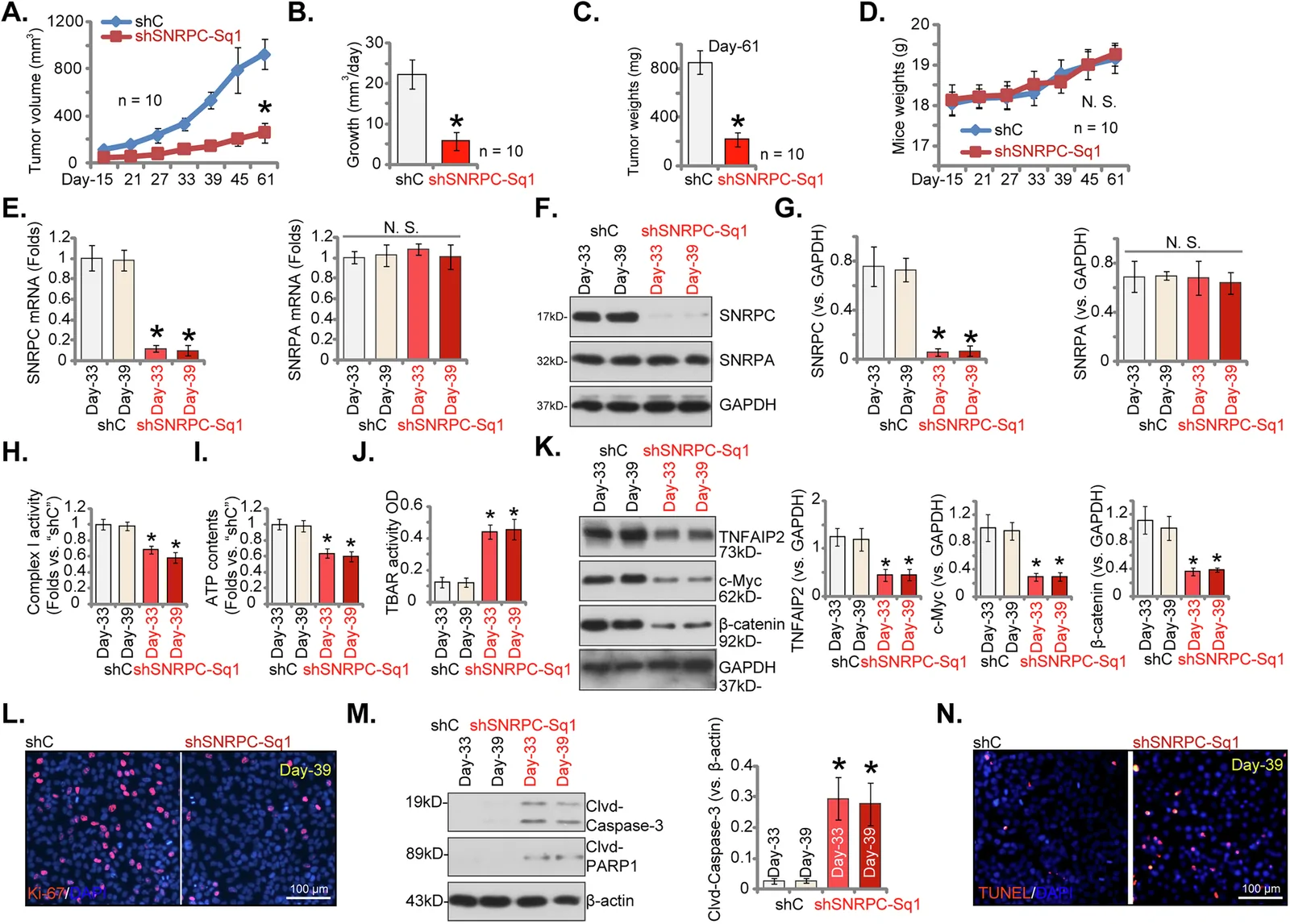
SNRPC knockdown suppresses subcutaneous glioma xenograft growth and disrupts mitochondrial bioenergetics. P1 glioma cells stably expressing shSNRPC-Sq1 or control shC were subcutaneously injected into nude mice, and tumor growth was monitored over 61 days, including measurement of tumor volume (**A**) and calculation of the tumor growth rate (**B**); at the experimental endpoint (Day-61), final tumor weights were measured (**C**), and the body weights of the mice were monitored (**D**). Excised tumor tissues were analyzed for *SNRPC* and *SNRPA* mRNA levels at Day-33 and Day-39 by qRT-PCR (**E**), and SNPRC protein knockdown was confirmed by Western blot analysis at the same time points (**F**, **G**); Mitochondrial function was assessed in tumor lysates by measuring ETC Complex I activity (**H**) and total ATP content (**I**), and oxidative stress was assessed by measuring TBAR activity for lipid peroxidation (**J**). TNFAIP2, c-Myc, and ß-catenin protein levels were tested via Western blotting assays (**K**). Immunofluorescence staining for the proliferation marker Ki67 was performed on the tumor sections (**L**); The apoptosis induction was confirmed by western blot analysis of cleaved Caspase-3 and cleaved PARP-1 in tumor lysates at Day-33 and Day-39 (**M**), and visually confirmed by TUNEL staining on tumor sections (**N**). Results are shown as mean ± standard deviation (SD). Data for **A**–**D** reflect *n* = 10 mice per group. For **E**–**N**, analyses utilized five distinct tissues randomly selected per xenograft (*n* = 5). Significance is denoted as *P < 0.05 versus the shC control group. “NS” denotes non-significance. Scale bar, 100 μm.

The necessity of SNRPC for tumor progression was validated in an in vivo subcutaneous xenograft model using P1 glioma cells stably expressing shSNRPC-Sq1 or control shC. This silencing using shSNRPC-Sq1 resulted in a highly significant suppression of tumor growth over the 61-day observation period (Fig. 10A). The calculated tumor growth rate was markedly diminished in the shSNRPC-Sq1 group (Fig. 10B). At the experimental endpoint (Day-61), mean tumor weights were drastically lower, reducing the xenograft mass from approximately 800 mg in the shC group to less than 200 mg in the knockdown tumors (Fig. 10C), with no discernible effect on the mice’s mean body weights (Fig. 10D). Molecular analysis of the excised tumors confirmed that the successful and sustained knockdown of *SNRPC* mRNA and protein persisted through the analysis at two different time points (Day-33 and Day-39) (Fig. 10E–G). *SNRPA* mRNA and protein levels were however unaffected (Fig. 10E–G). Consistent with the in vitro data, this sustained silencing of SNRPC led to profound mitochondrial dysfunction in vivo: tumors from the shSNRPC-Sq1 group exhibited a significant decrease in ETC Complex I activity (Fig. 10H) and a corresponding reduction in total ATP content (Fig. 10I). This bioenergetic disruption induced oxidative stress, indicated by a significant increase in TBAR activity (Fig. 10J), confirming elevated lipid peroxidation within the shSNRPC-Sq1 subcutaneous P1 tumors. Furthermore, Western blot analysis of SNRPC-silenced tumor lysates revealed a significant decrease in TNFAIP2, c-Myc, and ß-catenin protein levels (Fig. 10K), indicating downregulation of TNFAIP2-β-Catenin signaling.

The mechanism of tumor suppression was definitively linked to reduced cellular proliferation and increased apoptosis. Immunofluorescence staining for the proliferation marker Ki67 revealed a significant reduction in the proliferation rate within the shSNRPC-Sq1 tumors (Fig. 10L). Western blot analysis of tumor lysates confirmed apoptosis induction, showing a clear increase in the cleavages of Caspase-3 and PARP-1 at both Day-33 and Day-39 (Fig. 10M). This was visually corroborated by TUNEL staining, which demonstrated a significant increase in apoptotic cell nuclei in the knockdown tumors (Fig. 10N). These in vivo results unequivocally establish that SNRPC is critically required for sustaining mitochondrial metabolism, cellular proliferation, and driving the malignant progression of subcutaneous glioma xenograft.

### SNRPC knockout impedes intracranial glioma growth in an orthotopic mouse model

**Fig. 11:**
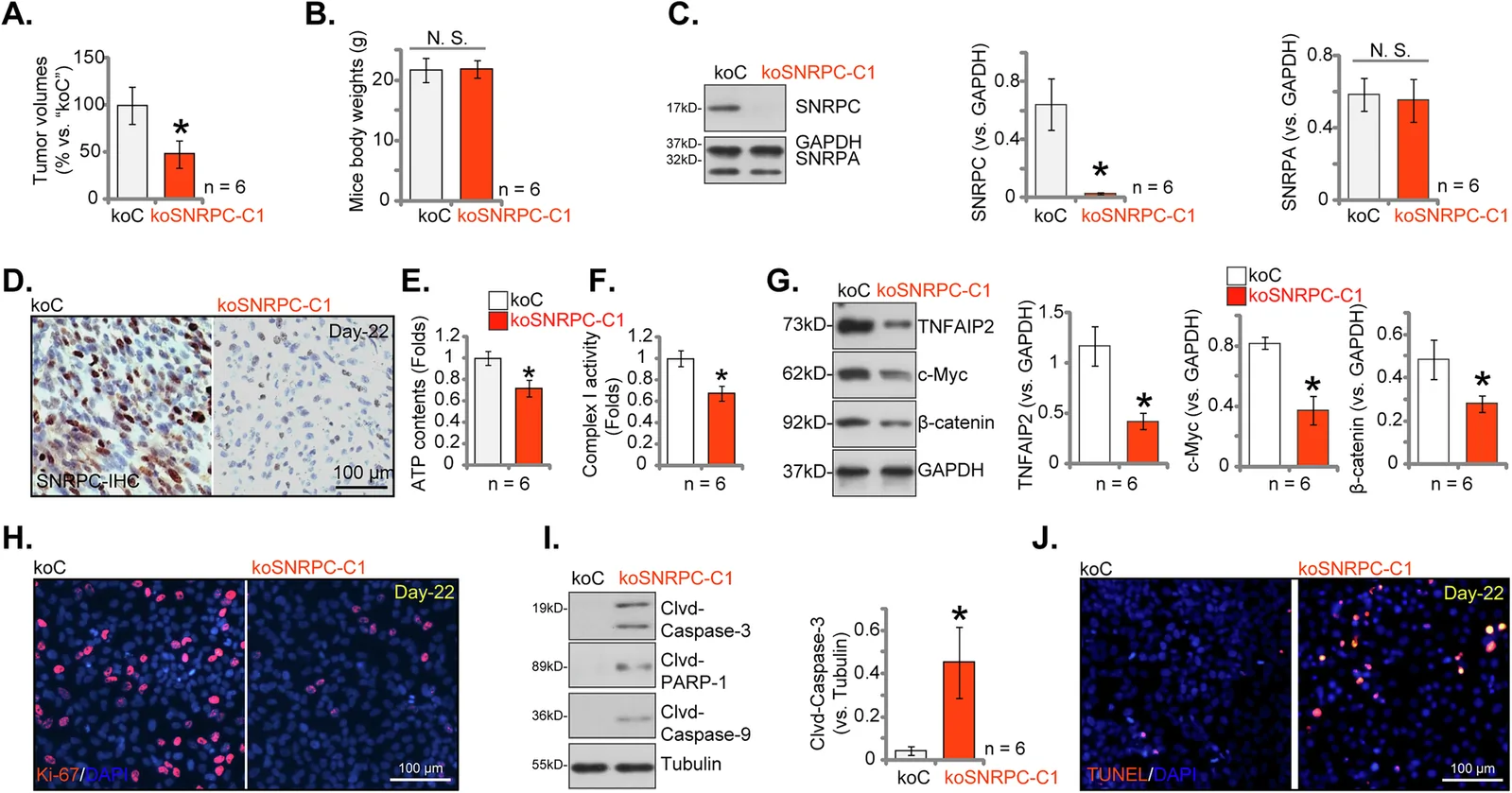
SNRPC knockout impedes intracranial glioma growth in an orthotopic mouse model. P1 glioma cells with stable SNRPC knockout (koSNRPC-C1) or control (koC) were stereotactically injected into the brains of nude mice for an orthotopic intracranial glioma model, and after 22 days, tumor growth was monitored by magnetic resonance imaging (MRI) and tumor volume quantification (**A**), with corresponding mouse body weights recorded (**B**). Excised intracranial tumor lysates were analyzed by Western blot to verify SNRPC protein depletion and stable SNRPA expression (**C**), and immunohistochemistry was performed to visually confirm SNRPC protein expression within the tumor sections (**D**). Bioenergetic function was assessed in tumor lysates by quantifying total ATP content (**E**) and ETC Complex I activity (**F**); TNFAIP2, c-Myc, and ß-catenin protein levels were tested via Western blotting assays (**G**). Cellular proliferation was determined by immunofluorescence staining for the marker Ki67 on tumor sections (**H**). Apoptosis was confirmed by Western blot analysis of cleaved Caspase-3, Caspase-9 and cleaved PARP-1 in tumor lysates (**I**), and visually validated by TUNEL staining on tumor sections (**J**). *n* = 6 mice per group. Significance is denoted as *P < 0.05 versus the koC control group. “NS” denotes non-significance. Scale bar, 100 μm.

At last, an orthotopic, intracranial glioma model was established. P1 glioma cells with stable SNRPC knockout (koSNRPC-C1) were stereotactically injected into the brains of nude mice and monitored against knockout control (koC) cells for 22 days. Magnetic resonance imaging (MRI) confirmed that SNRPC knockout profoundly suppressed tumor growth (Fig. 11A). Quantification of tumor volumes at the experimental endpoint (Day-22) revealed that tumor volumes in the koSNRPC-C1 were significantly lower compared to the koC group (Fig. 11A). This suppression of intracranial growth occurred without any significant difference in the body weights of the mice between the two groups (Fig. 11B). Molecular analysis of the excised intracranial tumors at the endpoint (Day-22) confirmed successful genetic ablation and recapitulated the central molecular mechanism observed in vitro. Western blot analysis verified the near-complete absence of SNRPC protein in the tumors, while expression of SNRPA remained stable (Fig. 11C). Immunohistochemistry visually reinforced this finding, showing minimal SNRPC staining in the SNRPC knockout tumors compared to the robust expression in controls (Fig. 11D). Importantly, koSNRPC-C1 tumors exhibited significant metabolic dysfunction: a marked reduction in total ATP content (Fig. 11E) and a commensurate decline in ETC Complex I activity (Fig. 11F). Analysis of intracranial koSNRPC-C1 tumor tissues revealed a significant reduction in the protein levels of TNFAIP2, c-Myc, and ß-catenin, confirming the inactivation of the TNFAIP2-ß-catenin signaling pathway in vivo (Fig. 11G). Furthermore, the immunofluorescence for the proliferation marker Ki67 revealed a significant reduction within the koSNRPC-C1 tumors (Fig. 11H). SNRPC knockout also induced apoptosis, evidenced by an increase in the cleavages of Caspase-3, Caspase-9 and PARP-1 (Fig. 11I) and visually confirmed by an increase in TUNEL-positive cells (Fig. 11J). These data provide compelling, definitive evidence that SNRPC is essential for the growth of glioma cells in the orthotopic environment.

## Discussion

The fidelity of pre-mRNA splicing, a process governing the expression of genes critical for cell functions, is a cornerstone of homeostasis; its dysregulation is increasingly recognized as an important driver of malignancy [18–20]. Within this context, SNRPC, a core component of the splicing machinery, has emerged as a molecule of significant interest in oncology. In TNBC cells, SNRPC maintains the structural integrity of the U1 snRNP complex to facilitate RNA Polymerase II enrichment at the promoters of key oncogenes like *E2F2*, *CDK4*, and *TNFAIP2*, thereby driving the transcriptional programs essential for malignant cell proliferation [20]. This pro-tumorigenic function extends to hepatocellular carcinoma (HCC). Zhang et al. documented a marked upregulation of SNRPC in HCC that corresponds with advanced tumor stages and unfavorable clinical outcomes [19]. Their work further revealed that SNRPC expression promotes cell motility and epithelial-mesenchymal transition (EMT) while being inversely correlated with immune cell infiltration, highlighting its dual impact on tumor [19]. Reinforcing these findings, Cai et al. confirmed that elevated SNRPC in HCC serves as a prognostic risk factor and is associated with lower stromal and immune scores [18]. A very recent study shows that overexpression of SNRPC drives chemoresistance in Wilms tumor by activating the NF-κB signaling pathway to induce CXCL17-mediated recruitment and polarization of M2-type tumor-associated macrophages [34]. In ovarian cancer, *SNRP*C was identified as an important RNA modification-related prognostic gene that is significantly upregulated in malignant tissues, effectively predicting clinical outcomes and sensitivity to chemotherapeutic agents like paclitaxel [35]. In endometrial cancer, SNRPC was identified as a key liquid-liquid phase separation (LLPS)-related biomarker that is significantly upregulated in tumor tissues and serves as a core component of a four-gene prognostic model used to identify high-risk patients with poor clinical outcomes and distinct immune microenvironment profiles [36].

The identification and validation of novel, functionally selective therapeutic targets are extremely important for improving patient outcomes. Our comprehensive analysis establishes SNRPC as both a robust prognostic biomarker and a promising therapeutic target in glioma. Bioinformatic analysis of the TCGA cohort revealed a highly significant, grade-dependent increase in *SNRPC* expression, peaking in the most aggressive WHO Grade IV tumors. This elevated expression consistently correlated with poor clinical outcomes, including reduced overall survival, progressive disease, and key molecular markers associated with a worse prognosis. Furthermore, single-cell RNA sequencing pinpointed SNRPC expression predominantly within the malignant glioma cell cluster, with the highest levels observed in highly aggressive recurrent GBM cells.

The functional requirement of SNRPC was unequivocally demonstrated through genetic manipulation in multiple primary and established glioma cell types. Selective silencing (shRNA) and definitive knockout (CRISPR/Cas9) of SNRPC severely attenuated proliferation, migration, and invasion, while inducing G1-S phase cell cycle arrest and apoptosis. Importantly, the anti-proliferative and pro-apoptotic effects of SNRPC depletion were strictly restricted to malignant glioma cells, having no discernible effect on normal human astrocytes. The specificity and oncogenic potential of SNRPC were further confirmed by its overexpression, which functionally augmented all tested malignant phenotypes. Importantly, the in vivo experiments showed that SNRPC knockdown or knockout significantly suppressed tumor growth in both subcutaneous xenograft and the clinically relevant orthotopic intracranial models.

A central characteristic of highly proliferative cancer cells, including high-grade glioma [32, 33], is their ability to sustain high levels of energy and biomass production, often driven by mitochondrial hyperfunction and enhanced OXPHOS. While historically the Warburg effect emphasized glycolysis, recent studies highlight the dependency of aggressive tumors, particularly those resistant to standard therapy, on robust mitochondrial respiration for ATP production, redox balance, and survival [32, 33]. This metabolic vulnerability offers a therapeutic window, as inhibiting mitochondrial hyperfunction can selectively induce bioenergetic crisis and apoptosis in cancer cells.

Our results strongly support that SNRPC is key for maintaining this mitochondrial hyperfunction in glioma. Bioinformatic analysis first established this link, showing that SNRPC co-expressed genes were highly enriched in terms such as cellular respiration, ETC, and OXPHOS. This prediction was experimentally validated: SNRPC silencing or knockout resulted in a profound bioenergetic collapse, marked by a decrease in basal and maximal OCR, reduced ETC Complex I activity, and significant depletion of cellular ATP content. This metabolic crisis led to immediate mitochondrial damage, demonstrated by the loss of mitochondrial membrane potential, followed by massive oxidative stress. Crucially, the resulting glioma cell apoptosis/death by SNRPC silencing could be significantly mitigated by supplementing cells with either the metabolic fuel (glucose) or the antioxidant (NAC), proving that the metabolic dysfunction and ensuing oxidative stress are causal components of the SNRPC depletion phenotype. These mechanistic findings were mirrored in vivo, where excised subcutaneous and intracranial glioma xenografts confirmed reduced ETC Complex I activity and ATP content following SNRPC silencing or knockout. Thus, SNRPC is an oncogenic driver in glioma that sustains the malignant phenotype by functionally maintaining mitochondrial hyperfunction and bioenergetic integrity.

TNFAIP2 has recently been identified as a pivotal regulator of oncogenic signaling pathways in cancer [37, 38]. Through its ability to upregulate the key oncogenes c-Myc and β-catenin, TNFAIP2 actively drives the initiation and malignant progression of TNBC [20]. c-Myc oncogene overexpression/overeactivation is a critical event in GBM, driving metabolic rewiring essential for tumor growth by controlling metabolic pathways and influencing key oncogene gene expression [39, 40]. Wnt/β-catenin pathway is a crucial therapeutic target in glioma due to its aberrant expression/activation and involvement in tumor progression, mediated by various molecular factors and crosstalk with PI3K/AKT signaling [41].

Our data indicate that SNRPC functions as a driver of glioma proliferation, mediated in part through the TNFAIP2-ß-catenin signaling axis. This conclusion is supported by transcriptional analysis revealing that the silencing or ablation of SNRPC in primary human glioma cells results in the downregulation of *TNFAIP2*, *CDK4*, and *E2F2*. Conversely, SNRPC overexpression was found to upregulate these transcripts. Notably, the inhibition of glioma cell growth caused by SNRPC knockdown was partially reversed by the restoration of TNFAIP2 expression, whereas the proliferative phenotype induced by SNRPC overexpression was abrogated by TNFAIP2 silencing. Furthermore, in vivo analysis of subcutaneous and intracranial glioma xenografts confirmed a significant reduction in TNFAIP2-β-catenin pathway components following SNRPC suppression. Collectively, these findings suggest that SNRPC promotes glioma tumorigenesis partly by activating the TNFAIP2-β-catenin signaling cascade.

In summary, this study identifies and validates SNRPC as a pivotal prognostic biomarker and a functionally indispensable driver of glioma malignancy. Our comprehensive analysis reveals a clear oncogenic addiction, whereby glioma cells are critically dependent on SNRPC for maintaining the mitochondrial hyperfunction and oncogenic TNFAIP2-ß-catenin signaling necessary for survival and proliferation. This dependency, which is absent in normal astrocytes and murine neurons, defines a clear therapeutic window and highlights a critical vulnerability. The strong correlation between SNRPC expression and poor clinical outcomes, combined with the potent anti-tumor efficacy of its depletion in preclinical models, firmly establishes its translational relevance. These findings warrant the prioritization of therapeutic strategies aimed at inhibiting SNRPC, offering a novel and mechanistically distinct approach to dismantle the oncogenic engine of glioma and possibly improve patient outcomes.

## Supplementary information

Figure S1-S2

Original Data Set

## Data Availability

All necessary data are provided in the paper and Supplementary Materials.
