# Supplementary material for: The spliceosome component SNRPC promotes glioma progression by sustaining mitochondrial function and TNFAIP2 signaling
Source: Cell Death Dis. 2026 Jul 17;17(1):883. doi: 10.1038/s41419-026-09065-6 (PMC13658826; doi:10.1038/s41419-026-09065-6)
Supplement: Supplementary file 1 [file 41419_2026_9065_MOESM1_ESM.pdf]

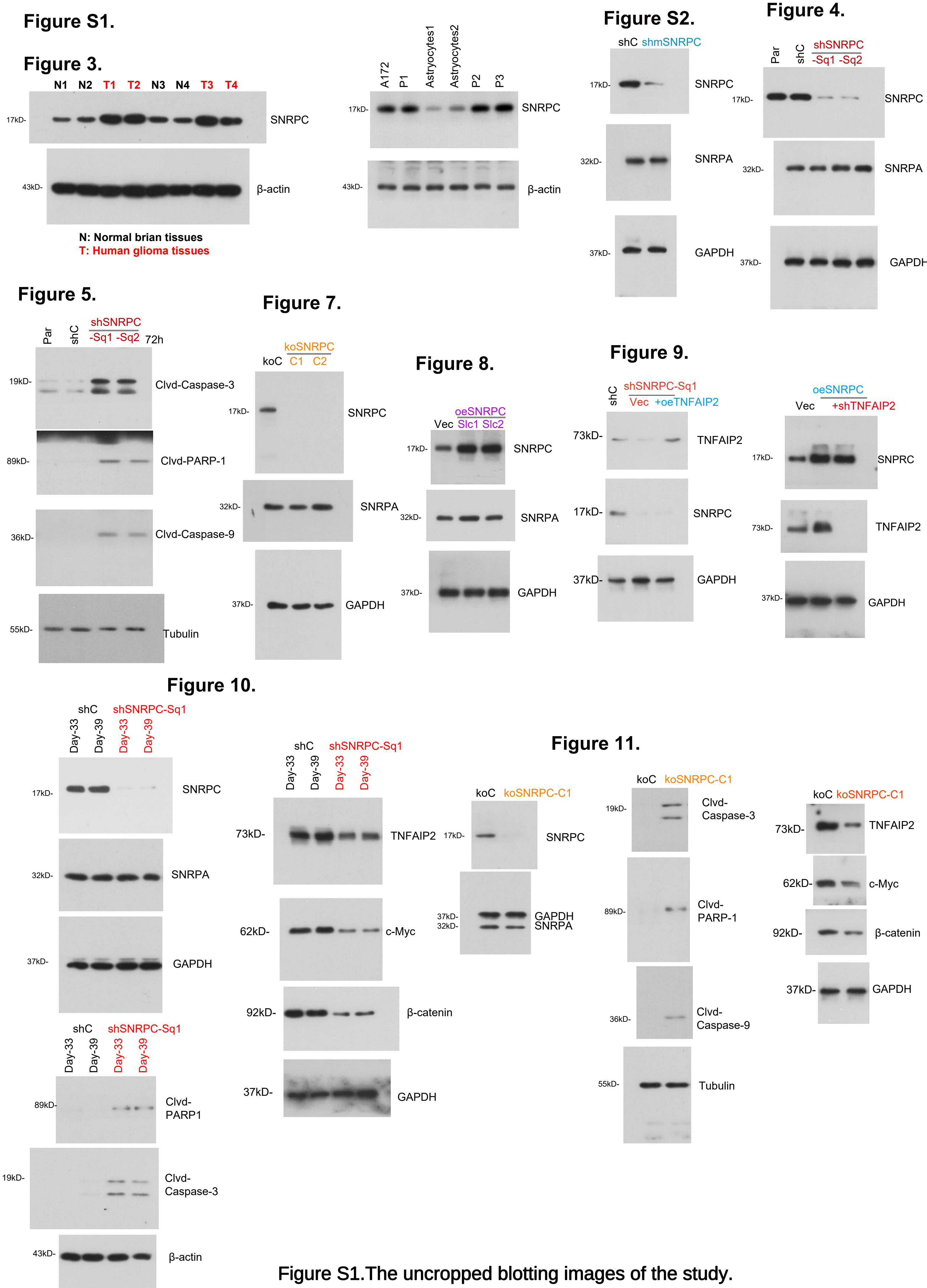

Figure S1.The uncropped blotting images of the study.

Figure S2.

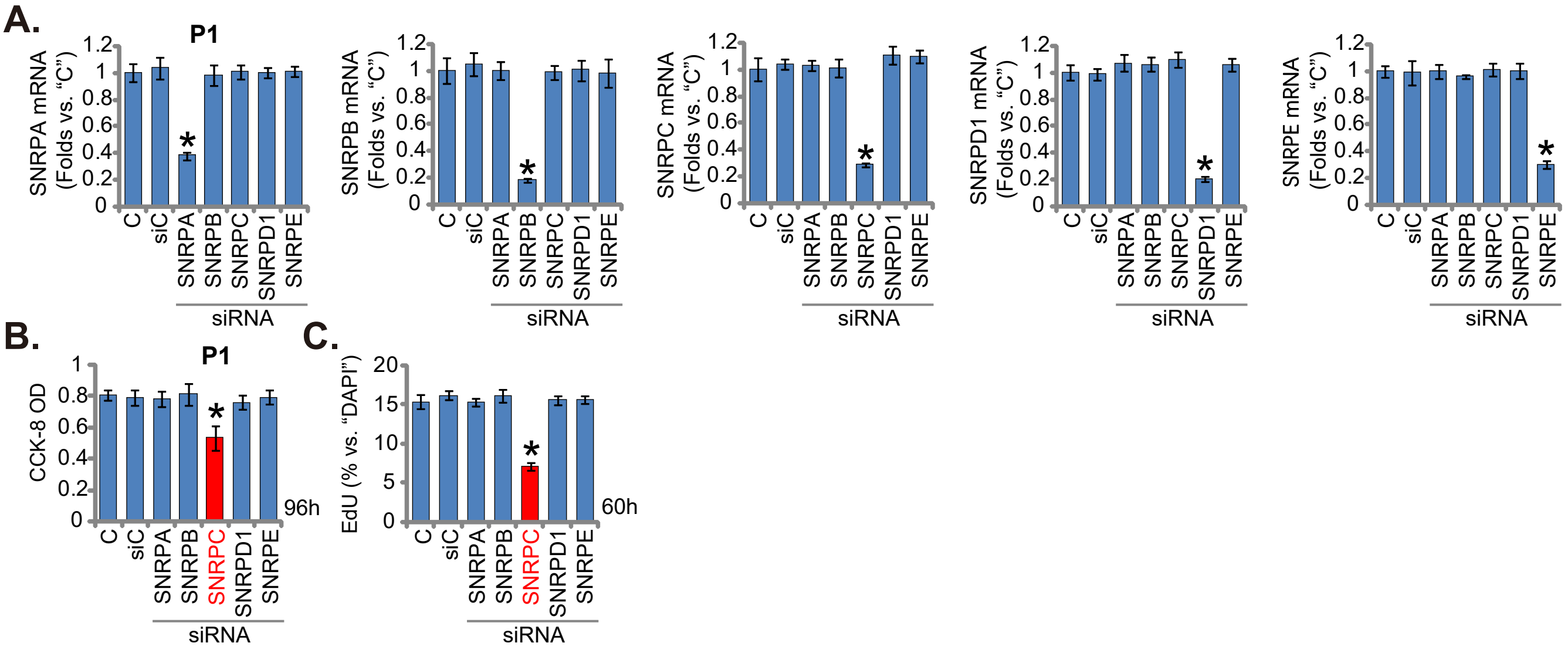

Figure S2. Functional screening of SNRP family components in P1 glioma cells. Quantitative RT-PCR (qRT-PCR) analysis of mRNA expression levels for *SNRPA*, *SNRPB*, *SNRPC*, *SNRPD1*, and *SNRPE* in P1 glioma cells following transfection with 200 nM of the indicated specific siRNAs (**A**). Transfections were performed twice at 24-hour intervals for a total of 48 hours, and mRNA levels are shown normalized to the control ("C") group (**A**). Cell viability assessed by CCK-8 assay at 96 hours post-transfection with the specified siRNAs (**B**). Cellular proliferation measured by EdU incorporation assay at 60 hours post-transfection (**C**). Data were presented as the percentage of EdU-positive cells relative to DAPI-stained total nuclei (**C**). Results are shown as mean ± standard deviation (SD) from five independent biological replicates (n=5). Significance is denoted as * ***P*** < 0.05 versus the siRNA control ("siC") cell group.

Figure S3.

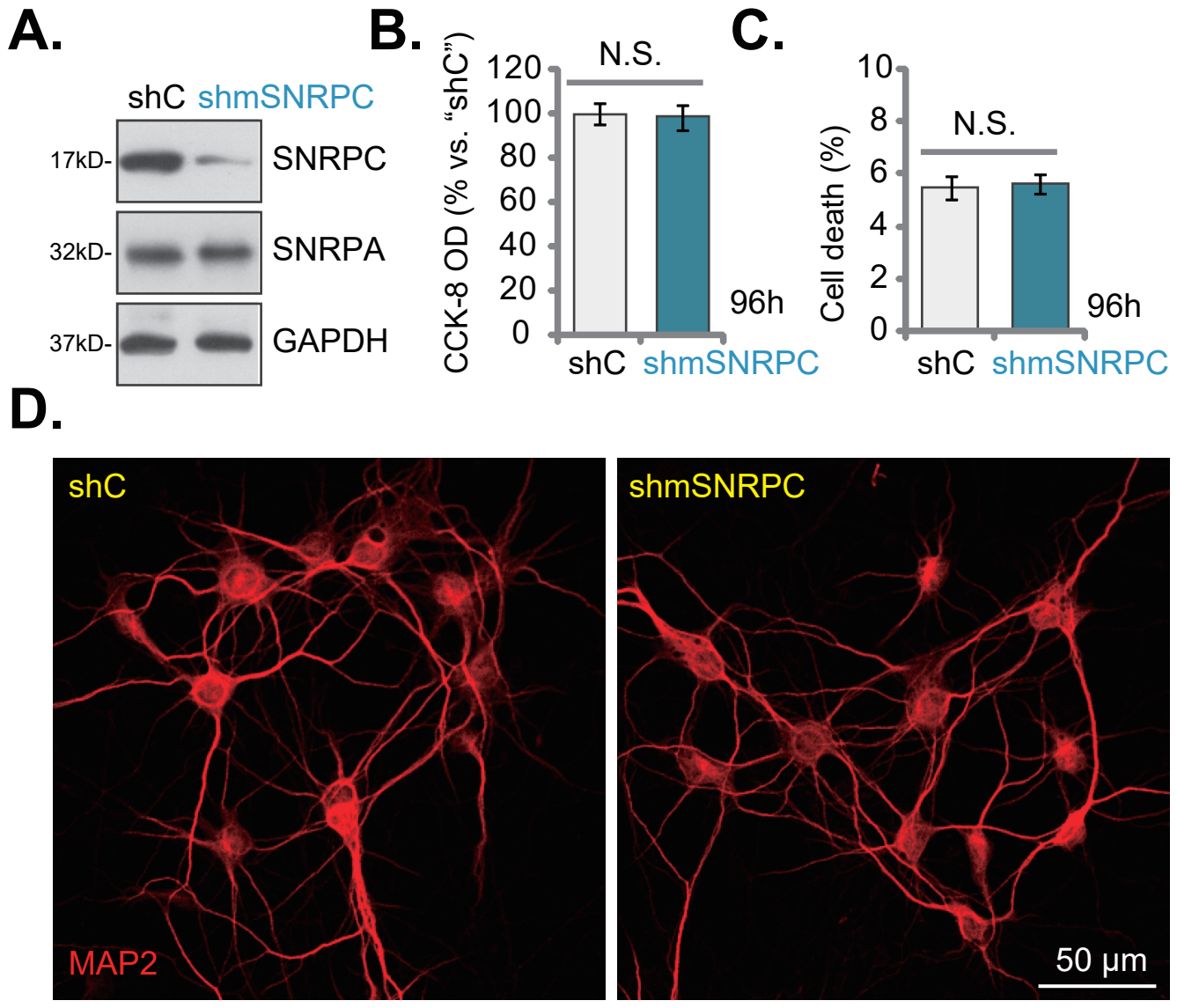

Figure S3. SNRPC and SNRPA protein expression in primary murine cortical neurons at P12 with the short hairpin RNA construct again murine SNRPC (shmSNRPC) or scrambled control shRNA (shC) was evaluated by Western blot analysis (**A**). After culture for indicated time periods, cell viability was assessed by the Cell Counting Kit-8 (CCK-8) assay (**B**), and the cell death rate was determined by the Trypan blue staining assay (**C**). Cell morphology was also shown using MAP2 immunofluorescence staining (**D**). Results are shown as mean ± standard deviation (SD) from five independent biological replicates (n=5). "N.S." denotes non-significance. Scale bar, 50 μm.
