# Supplementary material for: The spliceosome component SNRPC promotes glioma progression by sustaining mitochondrial function and TNFAIP2 signaling
Source: Cell Death Dis. 2026 Jul 17;17(1):883. doi: 10.1038/s41419-026-09065-6 (PMC13658826; doi:10.1038/s41419-026-09065-6)

Figure S1. The uncropped blotting images.

Figure 3.

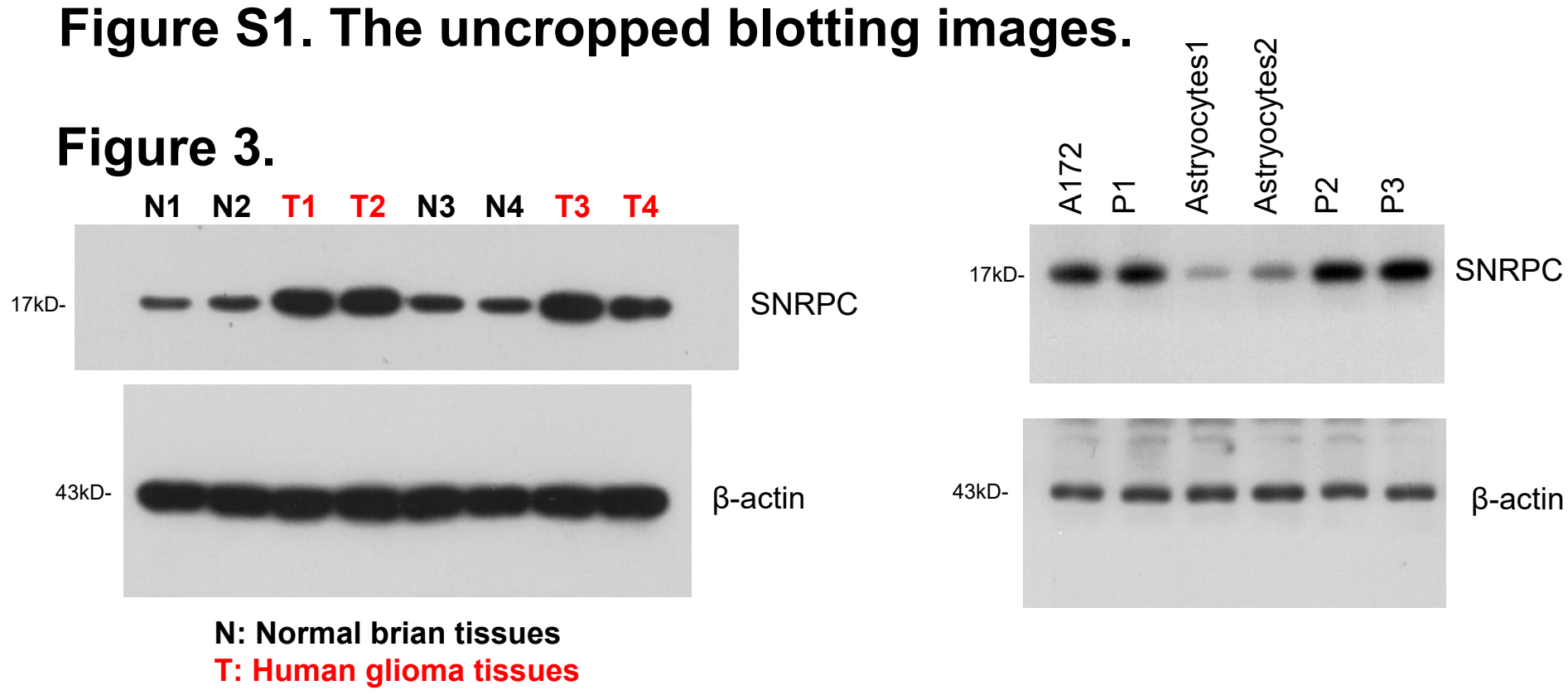

Figure S2.

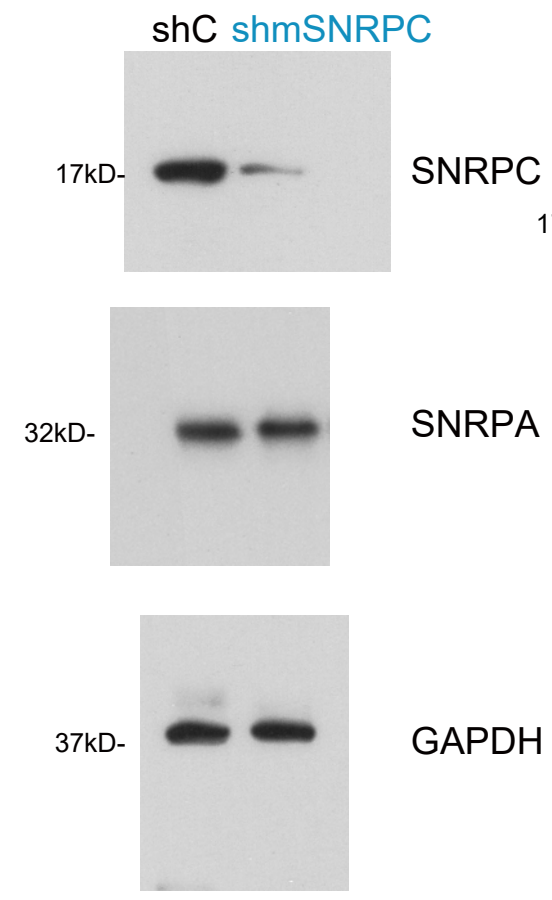

Figure 4.

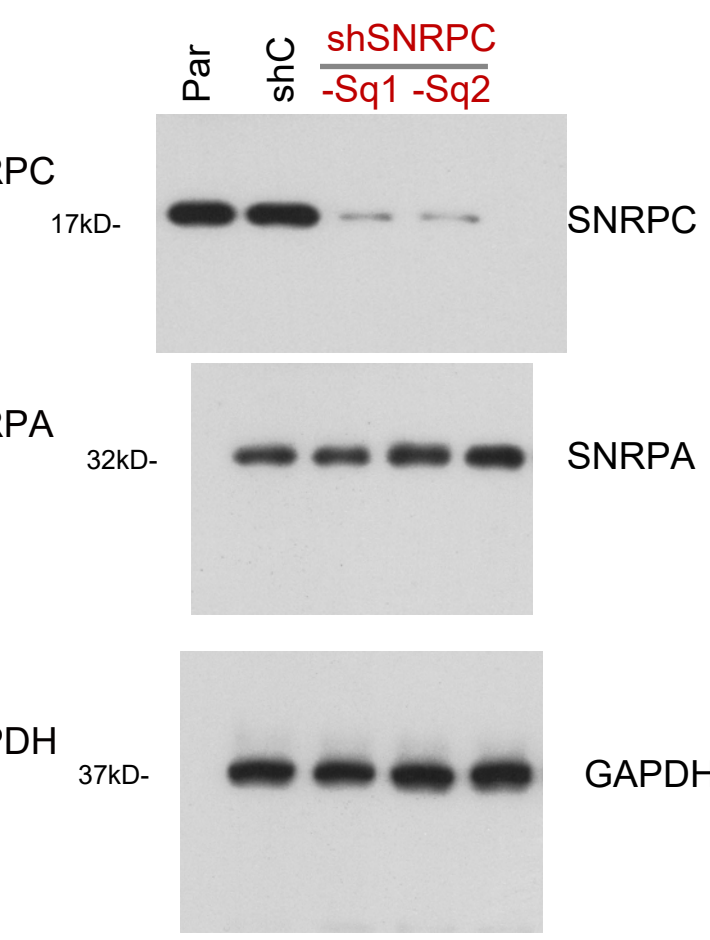

Figure 5.

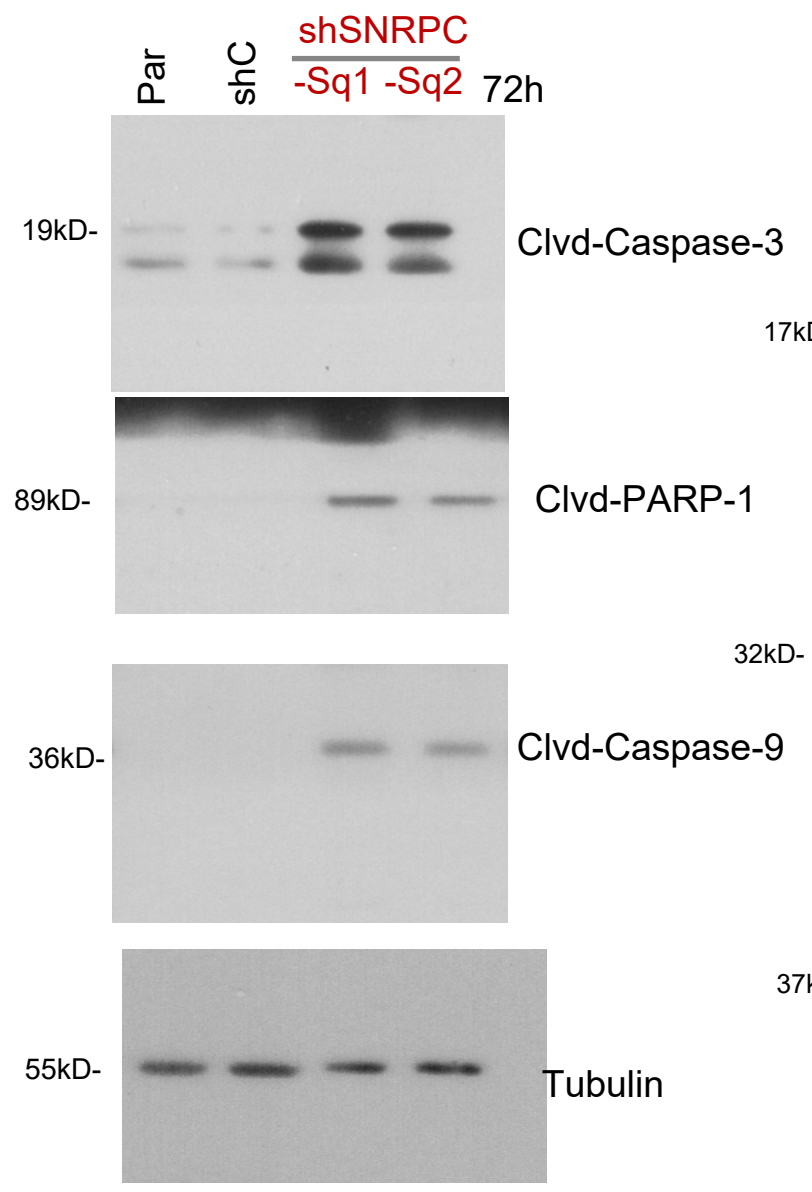

Figure 7.

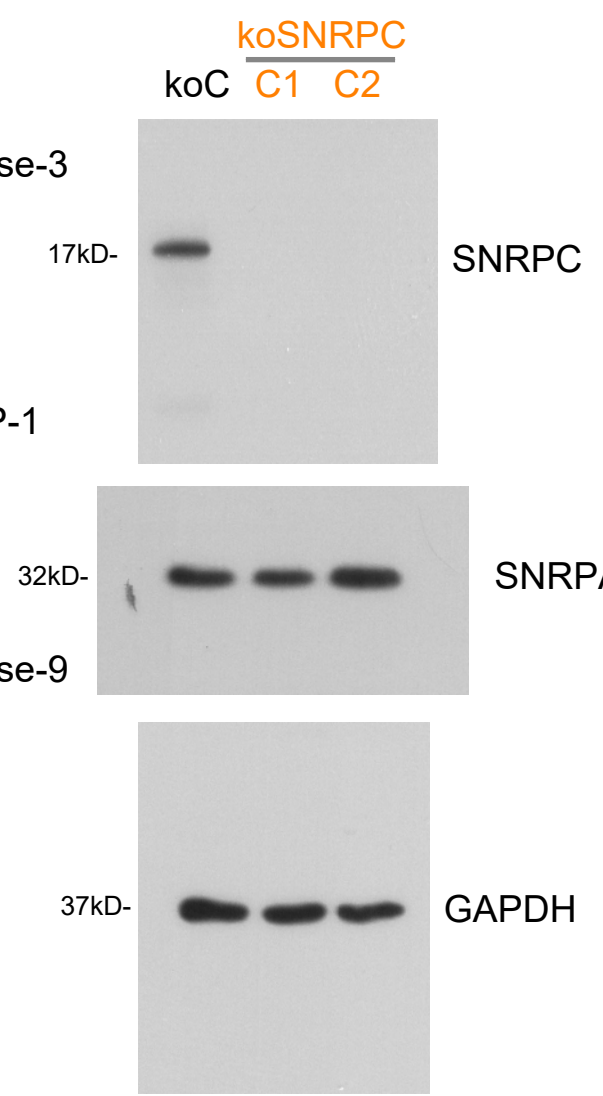

Figure 8.

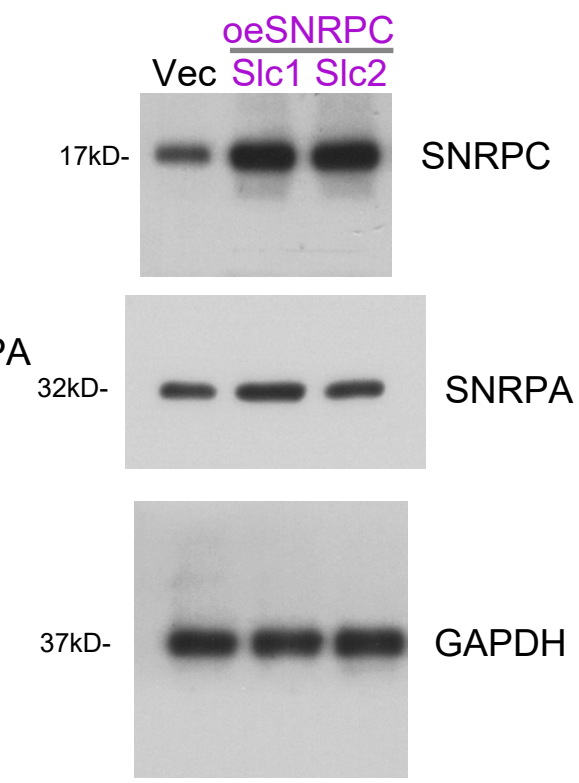

Figure 9.

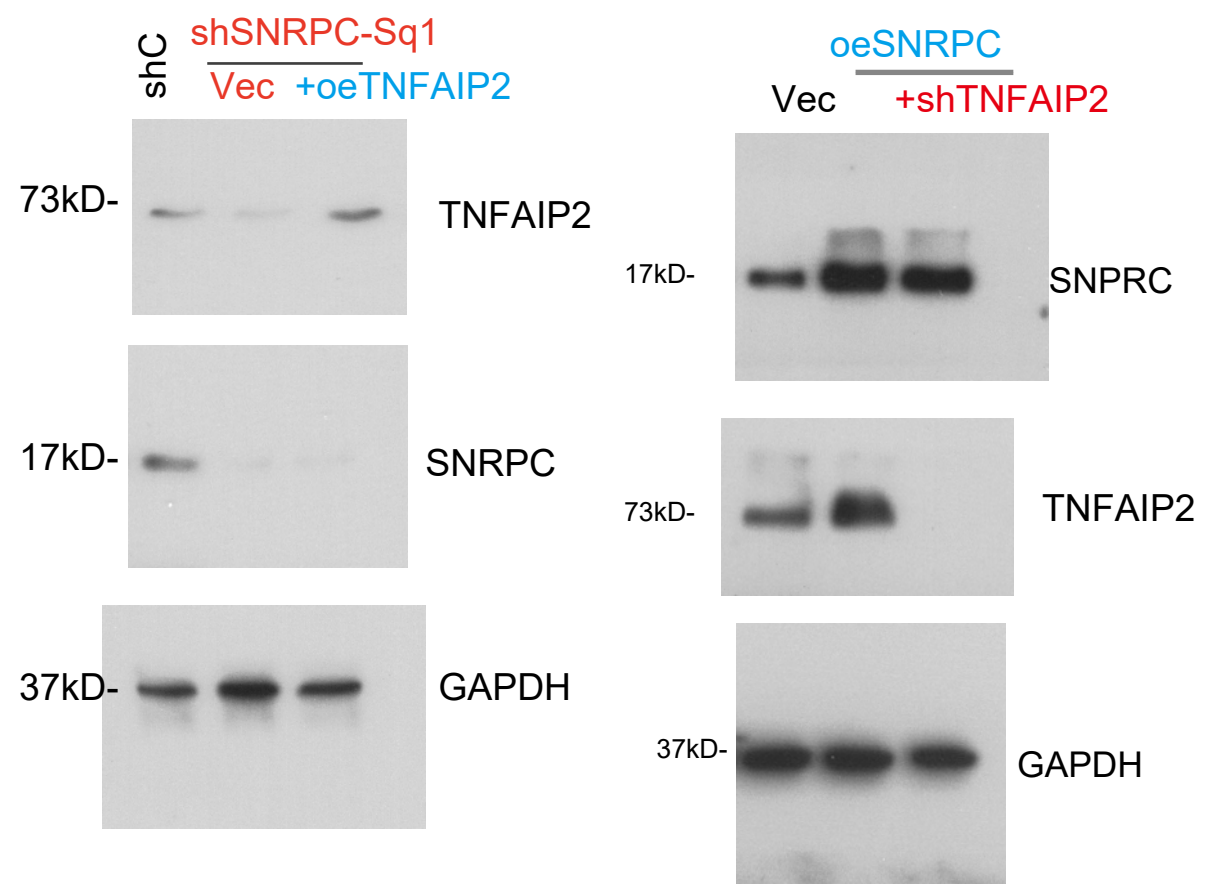

Figure 10.

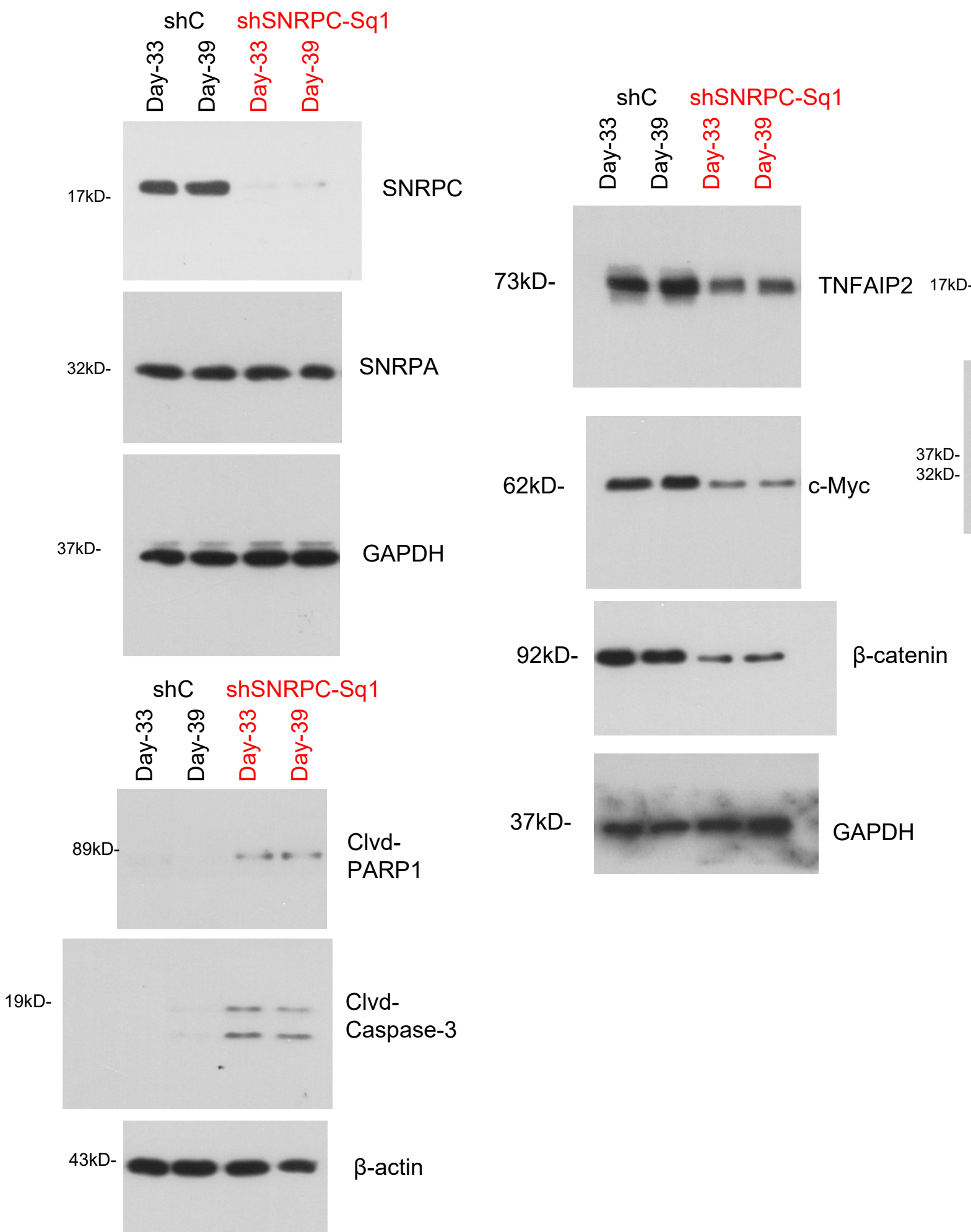

Figure 11.

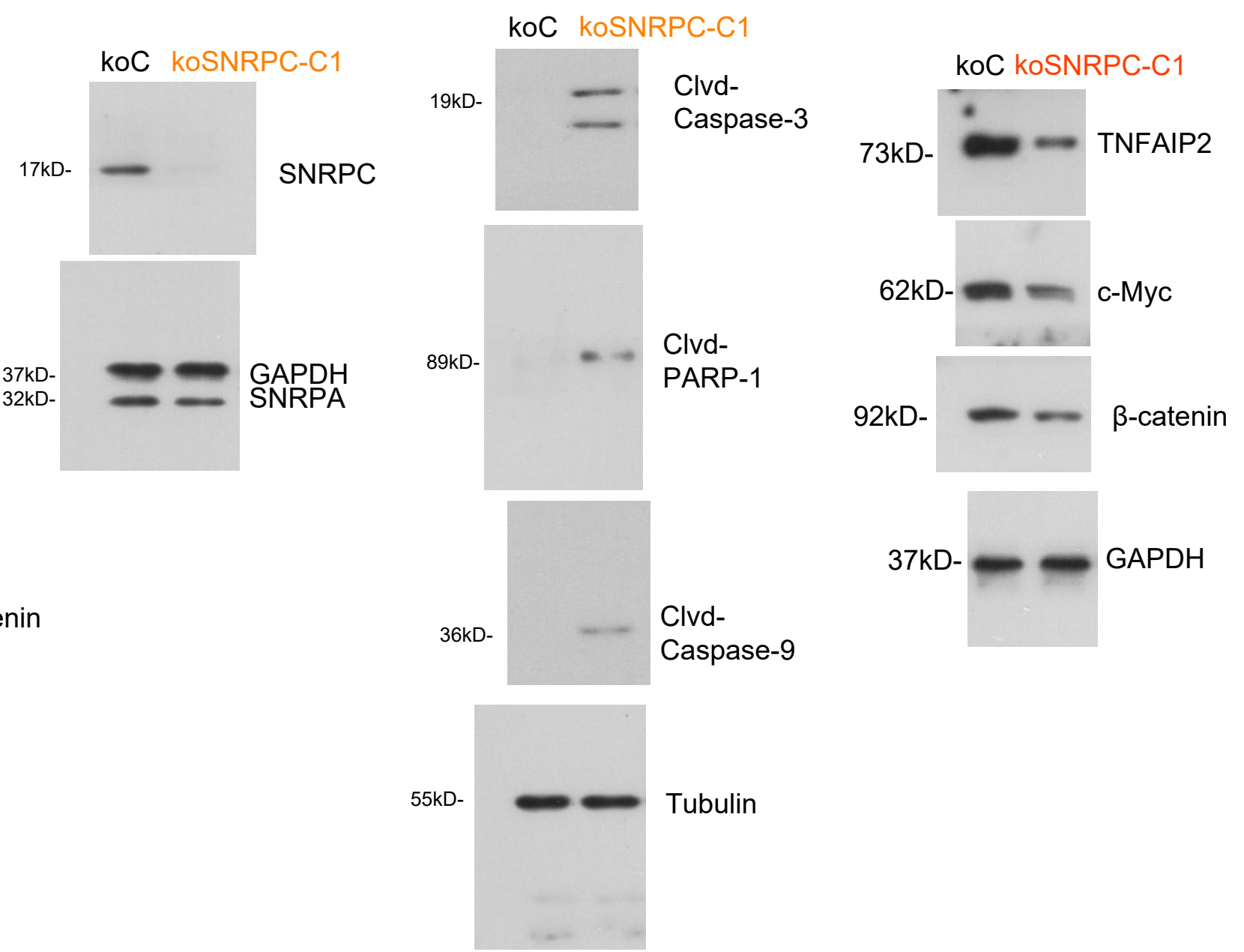

Supplement: Supplementary file 2 [file 41419_2026_9065_MOESM2_ESM.pdf]
